# An interpretable machine learning model for predicting postoperative hypotension in type 2 diabetes mellitus undergoing non‑cardiac surgery

**DOI:** 10.1186/s12933-026-03256-3

**Published:** 2026-06-22

**Authors:** Yu Gao, Guojiang Yin, Zheng Qi, Xiaoyang Song, Xiang Zhou, Kun Li

**Affiliations:** 1 General Hospital of Central Theater Command of People’s Liberation Army, Wuhan, China; 2 No. 991 Hospital of the Joint Logistics Support Force of the Chinese People’s Liberation Army, Xiangyang, China

**Keywords:** Postoperative hypotension, Machine learning, Type 2 diabetes mellitus, Explainable artificial intelligence, Random forest

## Abstract

**Background:**

Postoperative hypotension (POH) is a common and serious complication in patients with type 2 diabetes mellitus (T2DM) undergoing non‑cardiac surgery, yet predictive tools tailored to this high‑risk population remain scarce.

**Methods:**

This single‑center cohort study developed and validated a machine learning (ML) model to predict the risk of postoperative hypotension (POH) occurring during the post‑anaesthesia care unit (PACU) stay, defined as systolic blood pressure < 90 mmHg after leaving the operating theatre and before transfer to the general ward, consistent with the Perioperative Quality Initiative (POQI) consensus. Data from 34,012 retrospective (2012–2022) and 10,528 prospective (2023–2025) T2DM patients undergoing non‑cardiac surgery were used. Following rigorous preprocessing and a four‑step feature selection, 13 predictors were retained. Fourteen ML models were trained and evaluated using area under the curve (AUC), sensitivity, specificity, and calibration. Model interpretability was enhanced using SHapley Additive exPlanations (SHAP).

**Results:**

Random Forest achieved the best overall performance, with AUCs of 0.843 (95% CI 0.837–0.849) on training, 0.854 (95% CI 0.848–0.860) on internal validation, and 0.847 (95% CI 0.840–0.854) on prospective validation. External validation on an independent hospital cohort (*n* = 2156) yielded an AUC of 0.822 (95% CI 0.805–0.839), confirming generalisability. It demonstrated high sensitivity (0.932) and reliable calibration. SHAP analysis identified intraoperative blood loss, age, heart failure, obstructive sleep apnoea, and body mass index as the top predictors, providing transparent global and local explanations for individual risk.

**Conclusion:**

An interpretable ML model based on routinely collected clinical data accurately predicts POH risk in T2DM patients after non‑cardiac surgery. The model combines strong discriminative performance with clinical explainability, suggesting its potential as a practical tool for preoperative risk stratification and personalized postoperative monitoring in T2DM patients within similar clinical settings.

**Graphical abstract:**

**Figure:**
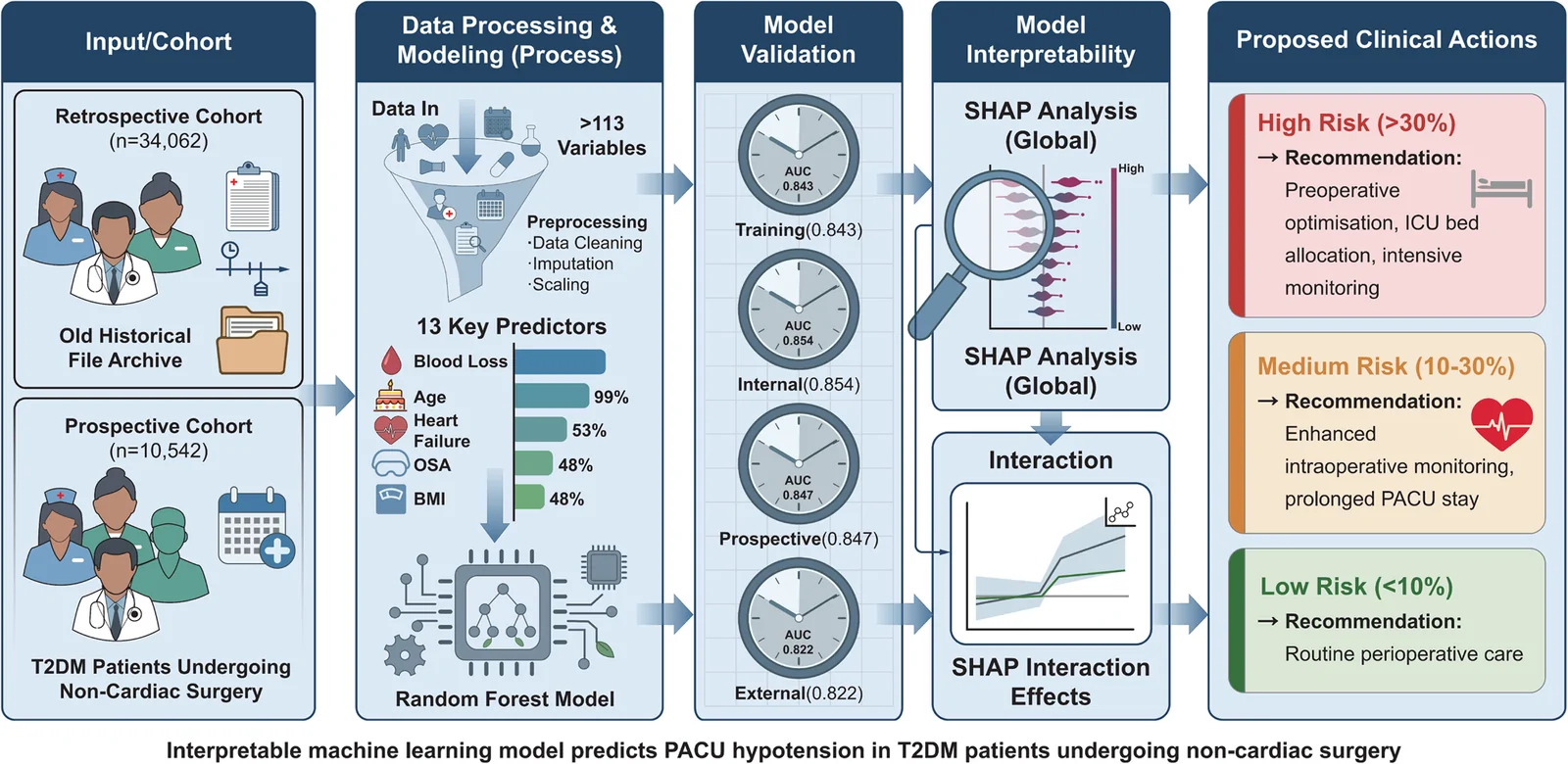

**Supplementary Information:**

The online version contains supplementary material available athttps://doi.org/10.1186/s12933-026-03256-3

## Research insights

**What is currently known about this topic?**

1. Postoperative hypotension is common and harmful in type 2 diabetes mellitus (T2DM) patients after non‑cardiac surgery.
2. T2DM patients have unique risks (e.g., autonomic dysfunction) for hemodynamic instability.
3. General machine learning models exist, but none are tailored for immediate postoperative hypotension occurring during the post‑anaesthesia care unit (PACU) stay in T2DM patients.

**What is the key research question?**

- Can an interpretable machine learning model accurately predict PACU‑based postoperative hypotension risk in T2DM patients?

**What is new?**

1. We developed a high‑performance, T2DM‑specific Random Forest model for PACU‑phase hypotension prediction.
2. Rigorous feature selection identified 13 actionable clinical predictors.
3. SHAP analysis provides transparent global and local explanations for model decisions.

**How might this study influence clinical practice?**

- It offers a practical tool for preoperative risk stratification and personalized postoperative monitoring (during PACU stay) in T2DM patients.

## Introduction

Postoperative hypotension (POH) is a common complication following surgery, with the risk of occurrence and adverse outcomes being particularly significant for patients with type 2 diabetes mellitus (T2DM) [1]. According to the Perioperative Quality Initiative (POQI) consensus, POH is specifically defined as a systolic blood pressure below 90 mmHg occurring after the patient leaves the operating theatre and before admission to a general ward [2]. This definition is adopted throughout the present study. Individuals with T2DM frequently present with multiple pathophysiological alterations, including autonomic dysfunction, vascular endothelial dysfunction, impaired volume regulation, and a chronic inflammatory state. These factors collectively compromise perioperative haemodynamic stability and compensatory capacity [3]. POH is independently associated with serious outcomes such as acute kidney injury, myocardial injury, stroke, and increased mortality [4]. For T2DM patients who already have underlying microvascular complications and diminished organ perfusion reserve, even relatively brief or mild reductions in blood pressure can trigger serious end-organ ischaemic events. Consequently, predicting the risk of POH in this patient population is of particular clinical urgency [5–6].

Although significant advances have been made in the monitoring and management of intraoperative hypotension [7–8], the recognition and intervention of POH remain major challenges after patients return to general wards, where monitoring is less intensive.Current commonly used clinical risk assessment tools, such as the Revised Cardiac Risk Index, rely primarily on a limited set of static clinical variables. They are thus insufficient for fully integrating and reflecting the complex clinical profile specific to T2DM patients [9–10]. Traditional statistical models, including logistic regression, also have inherent limitations when dealing with high-dimensional, non-linear relationships within large perioperative datasets [11–12]. The widespread adoption of electronic health records and advances in machine learning (ML) technology have made it possible to integrate and analyse multimodal clinical data [13–14]. ML has demonstrated superior performance in predicting intraoperative hypotension [15–16]. However, predictive models specifically focused on POH risk in the immediate postoperative period (PACU stay) for this high-risk T2DM population are still lacking. The development of such a model is crucial for enabling early warning, optimising resource allocation, and guiding individualised intervention [17].

Therefore, this study aims to develop and validate a machine learning model, using large-scale, single-centre perioperative data, specifically designed to predict the risk of POH following non-cardiac surgery in T2DM patients. We integrated multimodal data containing T2DM-related features and employed advanced algorithms capable of capturing complex non-linear relationships. To enhance the model’s clinical credibility and acceptability, we pursued high predictive performance while incorporating the SHapley Additive exPlanations (SHAP) framework for model interpretability analysis. This approach aims to provide transparent and intuitive insights by clearly revealing the specific contribution of each feature to the predicted risk at both global and individual levels, thereby supporting clinical decision-making [18–19].

## Materials and methods

### Study design and cohort establishment

This single-centre observational cohort study aimed to develop and validate a machine learning model to predict the risk of hypotension occurring during the post‑anaesthesia care unit (PACU) stay, i.e., after the patient leaves the operating theatre but before admission to the general ward. Data were extracted from the electronic health record (EHR), anaesthesia information management system (AIMS), and laboratory databases of the General Hospital of Central Theater Command (Wuhan, China), covering the period from January 2012 to September 2025.The study cohort was constructed according to explicit inclusion and exclusion criteria. Inclusion criteria required that patients met all of the following: (1) age ≥ 18 years; (2) undergoing non‑cardiac surgical procedures, including elective or emergency operations in general surgery, orthopaedics, urology, gynaecology, neurosurgery, or non‑cardiac thoracic surgery; (3) surgery performed under general anaesthesia (tracheal intubation or laryngeal mask airway) or combined general and regional anaesthesia; (4) postoperative transfer to a post‑anaesthesia care unit (PACU) for initial recovery, with planned subsequent admission to a general surgical ward; and (5) availability of complete and extractable perioperative records in the hospital’s electronic health record (EHR), anaesthesia information management (AIMS), and laboratory information systems, covering the key period from preoperative assessment until 48 h after surgery. Exclusion criteria comprised: (1) any cardiac surgical procedure (e.g., coronary artery bypass grafting, valve replacement); (2) a history of chronic hypotension, defined as a preoperative resting systolic blood pressure consistently < 90 mmHg or diastolic blood pressure consistently < 60 mmHg, or long‑term use of vasopressor medications for hypotension; (3) planned direct admission to an intensive care unit (ICU) after surgery or an expected ICU stay > 48 h; (4) total surgical duration < 30 min; (5) missing data exceeding 20% for core predictor variables; (6) pregnancy or lactation; and (7) coexisting severe end‑stage disease with a life expectancy < 30 days (e.g., New York Heart Association class IV heart failure, end‑stage renal disease requiring dialysis). Patients meeting these criteria formed the final study cohort.(8) Patients receiving pure regional anaesthesia (spinal anaesthesia or epidural anaesthesia as the primary anaesthetic technique) were not included in the study cohort, as per the inclusion criteria requiring general anaesthesia. Patients receiving combined general and regional anaesthesia were retained, as the predominant haemodynamic effects were attributable to general anaesthesia. (10) Preoperative chronic steroid use: defined as continuous daily use of prednisone ≥ 20 mg/day (or equivalent) for > 30 days prior to surgery. (11) Severe liver impairment: defined as cirrhosis Child‑Pugh class B or C, acute liver failure, or hepatic encephalopathy.The retrospective cohort (2012–2022, *n* = 34,012 after exclusions) was randomly split in a 7:3 ratio into a training set and an internal validation set. The prospectively collected cohort (2023–2025, *n* = 10,528 after exclusions) served as an independent temporal validation set.For external validation, we used an independent cohort from Hospital 991 (No. 991 Hospital of the Joint Logistics Support Force of the Chinese PLA), a tertiary centre 300 km away, with its own anaesthesia department, EHR system, and perioperative protocols. Inclusion/exclusion criteria were identical to the primary cohort. A total of 2156 T2DM patients undergoing non‑cardiac surgery under general anaesthesia (Jan 2023–Dec 2025) were included; 475 (22.0%) had PACU hypotension. No Hospital 991 data were used in model development; the locked random forest model was applied directly without retraining.This study adheres to the TRIPOD + AI (Transparent Reporting of a multivariable prediction model for Individual Prognosis Or Diagnosis – Artificial Intelligence extension) reporting guideline. A completed TRIPOD + AI checklist is provided in Supplementary File 1, with each item cross‑referenced to the relevant section of the manuscript.

### Data collection and variables

Data were extracted from the electronic health record (EHR), anaesthesia information management (AIMS), and laboratory systems. Variables collected included: demographic data (age, sex, body mass index, American Society of Anesthesiologists physical status classification); comorbidities; medication history; preoperative laboratory values; intraoperative data (vital signs, anaesthetic details, surgery type, blood loss, fluid administration); and the binary outcome (POH yes/no).

Through a comprehensive review of structured electronic medical records and anesthesia monitoring systems, a wide array of preoperative, intraoperative, and immediate postoperative variables was collected. The data encompassed the following domains:

#### Demographics

Age and gender.

#### Anthropometrics

Height, weight, Body Mass Index (BMI), Body Surface Area (BSA), and obesity grade.

#### Medical history & comorbidities

Medical history was extracted from electronic health records, including smoking history, American Society of Anesthesiologists (ASA) physical status classification, Charlson Comorbidity Index, and the presence of hypertension, coronary artery disease, congestive heart failure, chronic kidney disease, chronic obstructive pulmonary disease, obstructive sleep apnoea, arrhythmia, stroke, peripheral vascular disease, liver cirrhosis, autoimmune disease, and history of cancer.Diabetes severity indicators: In addition to the diagnosis of type 2 diabetes mellitus (T2DM), which was a universal inclusion criterion for the study, we collected markers reflecting the severity and chronicity of diabetes: Long‑standing diabetes: defined as a documented duration of T2DM > 10 years. Microvascular complications: defined as the presence of diabetic retinopathy (documented by ophthalmology consultation) or diabetic nephropathy (defined as persistent albuminuria > 30 mg/g creatinine or eGFR < 60 mL/min/1.73 m² attributed to diabetes). These two indicators were combined into a single binary variable, ‘Long‑standing diabetes (> 10 years) or microvascular complications’, which captures a higher risk profile for autonomic neuropathy and cardiovascular instability. This variable is distinct from the diagnosis of T2DM itself and is presented in Table 1 accordingly.Heart failure was identified from electronic health records based on documented clinical diagnosis (ICD-10I50). For patients with heart failure, New York Heart Association (NYHA) functional classification (I–IV) was recorded when available. In the retrospective cohort, NYHA classification was available for 4234 patients (55.4% of heart failure patients); in the prospective cohort, it was available for 1456 patients (64.9% of heart failure patients). Because NYHA documentation was incomplete, the primary model used a binary variable (presence/absence of heart failure). A sensitivity analysis was performed in the subset with NYHA data to compare model performance using binary heart failure versus ordinal NYHA classification; the results showed no significant difference (AUC 0.856 vs. 0.858, *P* = 0.32), confirming that the binary variable captures the predictive information adequately (Supplementary File 2). Steroid use in this study primarily reflects short‑term, low‑dose, or perioperative single‑dose administration. Chronic high‑dose users (prednisone ≥ 20 mg/day for > 30 days) were excluded. Cirrhosis primarily reflects compensated cirrhosis (Child‑Pugh class A). Decompensated cirrhosis (Child‑Pugh B/C, acute liver failure, hepatic encephalopathy) was excluded. Literature confirms that neither short‑term/low‑dose steroids nor compensated cirrhosis significantly increases postoperative hypotension risk [20–21].

**Table 1:** Baseline and preoperative characteristics of the retrospective development and prospective validation cohorts

Characteristic	Retrospective cohort (*N* = 34,012)			*P* value		Prospective cohort (*N* = 10,528)
Hypotension (*N* = 7822)	Non‑hypotension (*N* = 26,190)		Hypotension (*N* = 2219)	Non‑hypotension (*N* = 8309)		
*Demographics*						
Age, years	68.2 ± 9.8	61.8 ± 11.1	< 0.001	67.5 ± 10.2	60.9 ± 11.5	< 0.001
Male sex	4095 (52.4%)	13,756 (52.5%)	0.876	1166 (52.6%)	4279 (51.5%)	0.410
Body mass index, kg/m²	25.5 ± 3.8	24.6 ± 3.4	< 0.001	25.3 ± 3.6	24.5 ± 3.3	< 0.001
*Comorbidities*						
Hypertension	4312 (55.1%)	11,350 (43.3%)	< 0.001	1252 (56.4%)	3526 (42.4%)	< 0.001
Long‑standing diabetes (> 10 years) or microvascular complications*	3274 (41.9%)	8944 (34.2%)	< 0.001	952 (42.9%)	2854 (34.4%)	< 0.001
Coronary artery disease	2780 (35.6%)	6732 (25.7%)	< 0.001	810 (36.5%)	2042 (24.6%)	< 0.001
Congestive heart failure	2552 (32.6%)	5090 (19.4%)	< 0.001	744 (33.5%)	1498 (18.0%)	< 0.001
Chronic kidney disease	2336 (29.9%)	4966 (19.0%)	< 0.001	686 (31.0%)	1510 (18.2%)	< 0.001
Obstructive sleep apnoea	831 (10.6%)	2090 (8.0%)	< 0.001	256 (11.5%)	755 (9.1%)	< 0.001
History of stroke	811 (10.4%)	1991 (7.6%)	< 0.001	234 (10.5%)	656 (7.9%)	< 0.001
History of cancer	1170 (15.0%)	4270 (16.3%)	0.103	347 (15.7%)	1292 (15.6%)	0.936
Liver cirrhosis	462 (5.9%)	1233 (4.7%)	< 0.001	134 (6.0%)	421 (5.1%)	0.120
*Preoperative medication*						
Beta‑blocker	2476 (31.7%)	6650 (25.4%)	< 0.001	711 (32.0%)	2071 (24.9%)	< 0.001
ACEI/ARB	2746 (35.1%)	7456 (28.5%)	< 0.001	777 (35.0%)	2339 (28.2%)	< 0.001
Calcium channel blocker	1482 (19.0%)	3958 (15.1%)	< 0.001	421 (19.0%)	1264 (15.2%)	< 0.001
Diuretic	2116 (27.1%)	5200 (19.9%)	< 0.001	611 (27.5%)	1606 (19.3%)	< 0.001
Insulin	1872 (23.9%)	4933 (18.8%)	< 0.001	533 (24.0%)	1587 (19.1%)	< 0.001
Antiplatelet agent	1952 (25.0%)	5550 (21.2%)	< 0.001	555 (25.0%)	1709 (20.6%)	< 0.001
Anticoagulant	2146 (27.4%)	6358 (24.3%)	< 0.001	611 (27.5%)	2027 (24.4%)	< 0.001
Steroid	802 (10.3%)	2025 (7.7%)	< 0.001	234 (10.5%)	777 (9.4%)	0.145
*Preoperative vital signs*						
Systolic BP, mmHg	122 ± 15	130 ± 16	< 0.001	123 ± 14	131 ± 15	< 0.001
Diastolic BP, mmHg	72 ± 10	77 ± 10	< 0.001	73 ± 9	78 ± 10	< 0.001
Heart rate, bpm	82 ± 13	77 ± 11	< 0.001	81 ± 12	76 ± 10	< 0.001
*Preoperative laboratory*						
Hemoglobin, g/dL	11.9 ± 1.7	13.1 ± 1.7	< 0.001	12.1 ± 1.6	13.3 ± 1.6	< 0.001
Hematocrit, %	36.0 ± 5.0	39.3 ± 5.0	< 0.001	36.5 ± 4.8	39.7 ± 4.8	< 0.001
Creatinine, mg/dL	1.3 [1.0, 1.7]	1.0 [0.8, 1.3]	< 0.001	1.2 [1.0, 1.6]	1.0 [0.8, 1.2]	< 0.001
eGFR, mL/min/1.73 m²	65.1 ± 23.8	74.8 ± 23.5	< 0.001	67.6 ± 22.9	77.8 ± 22.8	< 0.001
Albumin, g/dL	3.6 ± 0.6	3.8 ± 0.5	< 0.001	3.6 ± 0.5	3.9 ± 0.5	< 0.001
Fasting glucose, mg/dL	158 [128, 195]	141 [115, 177]	< 0.001	155 [126, 190]	139 [113, 175]	< 0.001

Long‑standing diabetes (> 10 years) or microvascular complications: a composite variable indicating either diabetes duration exceeding 10 years or the presence of diabetic retinopathy/nephropathy. After exclusion of 50 retrospective and 14 prospective patients with chronic high‑dose steroid use or decompensated liver disease (exclusion rate < 0.2%). Statistical methods: Continuous variables are presented as mean ± SD (normal) or median [IQR] (non‑normal). Comparisons: t‑test or Mann‑Whitney U test; categorical variables: chi‑square test (or Fisher’s exact). All *p* values two‑tailed.

#### Preoperative medication

Use of ACEI/ARBs, beta-blockers, calcium channel blockers, diuretics, insulin, antiplatelet agents, anticoagulants, and steroids.

#### Preoperative status

Vital signs (SBP, DBP, MAP, HR, RR, SpO₂) and laboratory values (Hemoglobin, Hematocrit, Creatinine, eGFR, BNP, Albumin, Sodium, Potassium, Glycated haemoglobin (HbA1c, %)).

#### Surgical & anesthetic details

Emergency surgery status, surgery and anesthesia duration, estimated blood loss, transfusion requirement, and volumes of crystalloid and colloid administered.Surgical type: Categorised by specialty (general surgery, orthopaedics, urology, etc.). Surgical complexity: Work relative value units (Work RVU) obtained from the 2025 Medicare Physician Fee Schedule; surgical duration also recorded.

#### Intraoperative hemodynamics & monitoring

Key parameters included minimum MAP, hypotension time and area-under-the-curve (AUC) of hypotension, blood pressure and heart rate variability, Stroke Volume Variation (SVV), Pulse Pressure Variation (PPV), vasopressor use time and maximum dose, and urine output.Vasopressor use: defined as administration of any vasoactive agent (norepinephrine, epinephrine, dopamine, vasopressin, etc.). Dose standardised to norepinephrine equivalents (mcg/kg/min) using established conversion formulas. Cumulative duration recorded (minutes). Total dose and average infusion rate calculated.Calculated as the integral of the difference between the SBP threshold (90 mmHg) and the measured SBP over time during hypotensive episodes, expressed in mmHg·min. This parameter is distinct from the model performance metric AUC (area under the ROC curve).PPV and SVV were available only for patients with invasive arterial monitoring (retrospective: 42.2%, prospective: 43.5%). PPV was automatically calculated from the arterial waveform by the anaesthesia monitor. SVV was measured using the Flotrac/Vigileo system or oesophageal Doppler. Missing values were handled by multiple imputation (MICE, 20 imputations). Complete‑case analysis (invasive monitoring only) yielded an AUC of 0.859, compared to 0.853 with imputation, confirming no substantial bias (Supplementary File 3).

#### Anesthesia depth & brain monitoring

Bispectral Index (BIS) metrics (mean, min, time outside range, variability), Spectral Edge Frequency (SEF95), Burst Suppression Ratio (BSR), and Patient State Index (PSI).

#### Anesthetic agents

Doses of etomidate, rocuronium, sufentanil, propofol, remifentanil; mean end-tidal anesthetic agent (ETAA) concentration; Target-Controlled Infusion (TCI) level; MAC-hours; and use of reversal agents (sugammadex, atropine, flumazenil).

#### Intraoperative physiological metrics

Minimum temperature, hypothermia duration, minimum EtCO₂ and SpO₂, glucose range, peak lactate, minimum pH, and minimum base excess.

#### Postoperative recovery (PACU)

To address the reviewer’s concern about information leakage (variables measured after the outcome could bias predictions), we restricted this section to variables available before or at the time of the outcome. Data collected during the PACU stay included vital signs at PACU admission (systolic and diastolic blood pressure, heart rate, respiratory rate, oxygen saturation), changes in systolic blood pressure and heart rate within the first hour after admission, pain score at admission (0–10 numerical rating scale), presence of shivering, and level of respiratory support required (room air, nasal cannula, face mask, non‑invasive ventilation, or invasive ventilation). Variables measurable only after the outcome (e.g., 3‑hour fluid balance, 4‑hour urine output, 6‑hour lactate clearance) were excluded to prevent information leakage (see “Temporal ordering of predictors” section).Variables measured at 3, 4, and 6 h were excluded from the candidate predictor pool to prevent information leakage (see “Temporal ordering of predictors” section). They are reported here only for descriptive completeness.

#### Postoperative outcomes

Need for vasopressors, occurrence of postoperative nausea and vomiting (PONV), shivering, level of respiratory support required, and use of patient-controlled analgesia (PCA).

#### Outcome definition

ostoperative hypotension (POH) was defined as a systolic blood pressure < 90 mmHg recorded at any time during the PACU stay(from PACU admission to ward transfer). Consistent with the POQI consensus [4], this definition includes all hypotensive events from arrival in the PACU until the moment of transfer to the general ward. Patients with multiple hypotensive episodes were considered as having the outcome (binary: yes/no). The outcome was ascertained from electronic nursing records and vital signs monitors, with all measurements verified by two independent research assistants.

#### Temporal ordering of predictors

All candidate predictor variables were selected based on their temporal availability relative to the outcome (POH during PACU stay). Specifically, we included only variables that could be obtained before or at the time of PACU admission (e.g., preoperative, intraoperative, and immediate PACU admission data), ensuring that no information from after the outcome (i.e., after the patient had already experienced hypotension) was used for prediction. Any variable that could only be measured after the outcome had occurred (e.g., 3‑hour fluid balance, 4‑hour urine output, 6‑hour lactate clearance, 6‑hour haemoglobin change, postoperative respiratory rate measured after ward transfer) was excluded from the candidate pool.A comprehensive definition of all variables, including units, measurement timing, and data sources, is provided in the Supplementary Table S1.

### Data preprocessing and feature selection

Missing continuous data (< 5%) were imputed using the median; categorical data underwent mode imputation. For variables with partial availability (e.g., PPV and SVV, which were only recorded for patients with invasive arterial monitoring), we used multiple imputation by chained equations (MICE) with 20 imputations. The imputation model included all candidate predictor variables as well as the outcome, and we pooled performance metrics across imputed datasets using Rubin’s rules. This approach minimises bias while preserving sample size (Supplementary File 3). Outliers were winsorised based on the interquartile range (IQR) rule. Continuous variables were standardised using Z‑scores. From an initial set of over 113 variables, a four‑step feature selection process was employed: (1) univariate logistic regression (*P* < 0.05); (2) variance inflation factor (VIF) analysis (VIF < 10); (3) importance ranking via eight algorithms (e.g., Boruta, XGBoost); and (4) recursive feature elimination (RFE) to optimise sensitivity, specificity, and the area under the curve (AUC). This process yielded 13 final predictors.Throughout the feature selection process, we strictly adhered to the temporal constraint described in “Data collection and variables” section. This approach eliminates the risk of information leakage and ensures that the final model reflects a clinically realistic scenario—predicting POH based on data available before the outcome occurs.All feature selection steps—including univariate logistic regression (*p* < 0.05), variance inflation factor (VIF) analysis (VIF < 10), multi‑algorithm importance ranking (using Boruta, XGBoost, random forest, LASSO, GBDT, KNN, neural network, and logistic regression), and recursive feature elimination (RFE)—were performed exclusively on the training set using 10‑fold cross‑validation. The internal validation set and the prospective validation set were not used in any step of feature selection. The final feature subset was determined based on the performance observed in the training cross‑validation, and then locked before any evaluation on the validation sets.

### Model development and training

Fourteen machine learning models were trained using 10‑fold cross‑validation to maximise AUC, including linear (logistic regression, LASSO), tree‑based (random forest), gradient boosting (XGBoost, GBDT, AdaBoost), KNN, naïve Bayes, neural networks, and stacking ensembles. The selection aimed to systematically explore which algorithmic family best suits this clinical context; rather than hypothesis testing, we conducted model selection by evaluating algorithms across different paradigms. Performance was compared using multiple metrics (AUC, sensitivity, specificity, Brier score, calibration, decision curve analysis) to avoid reliance on a single measure. Hyperparameters were tuned via grid search with cross‑validation; final parameters were selected based on highest mean AUC (simpler model chosen in case of ties). All models were implemented in R using `caret`, `randomForest`, `xgboost`, `glmnet`, `kernlab`, and `naivebayes`. Stacking ensembles used logistic regression as the meta‑learner, with base learner predictions generated via cross‑validation to prevent overfitting.

### Model evaluation and interpretability

Models were evaluated on the training (with cross‑validation), internal validation, and prospective validation cohorts using the area under the curve (AUC), accuracy, sensitivity, specificity, precision, F1‑score, Brier score, and calibration curves. Interpretability of the best‑performing model was analysed using SHapley Additive exPlanations (SHAP) to provide both global and local explanations.95% confidence intervals (CIs) for all performance metrics were calculated using bootstrap resampling with 1000 replicates. For each replicate, we randomly sampled the validation set with replacement, computed the metric, and recorded the value. The 2.5th and 97.5th percentiles of the bootstrap distribution were taken as the 95% CI.

### Model interpretability analysis

To enhance the interpretability of the optimal machine learning model, we applied the SHapley Additive exPlanations (SHAP) framework at both global and local levels. Global interpretation employed SHAP summary and beeswarm plots to visualise the ranking of feature importance and the direction of each feature’s contribution to predicted risk. Local interpretation used SHAP force and waterfall plots to illustrate how individual features shifted a patient’s predicted risk from the baseline, thereby providing case‑specific decision support for clinicians.

### Statistical analysis plan

A detailed statistical analysis plan (SAP) was pre‑specified before data analysis and is available in Supplementary File 4 (Statistical Analysis Plan). Briefly, the SAP outlines the approach to data preprocessing (missing data imputation, outlier treatment, standardisation), feature selection (univariate screening, VIF analysis, multi‑algorithm importance ranking, recursive feature elimination with cross‑validation), model training and validation (14 models, 10‑fold cross‑validation, hyperparameter tuning), performance metrics (area under the curve, sensitivity, specificity, Brier score, calibration, decision curve analysis), and SHapley Additive exPlanations (SHAP) for model interpretability (global and local explanations). Pre‑specified subgroup analyses (elective vs. emergency surgery, presence of invasive monitoring, heart failure status) are also detailed. All analyses were conducted in accordance with this pre‑specified plan.

### Software and statistical tools

All data preprocessing, feature engineering, machine learning modelling, evaluation, and interpretability analyses were conducted using R (version 4.5.2). Key packages utilised included: tidyverse and ggplot2 for data manipulation and visualisation; caret, randomForest, xgboost, glmnet, kernlab, and naivebayes for modelling and cross‑validation; Boruta and RFE for feature selection; shap for SHapley Additive exPlanations (SHAP)‑based interpretability; and pROC, Resource Selection, and rmda for statistical and calibration assessment.

## Results

### Baseline characteristics of patient cohorts

Baseline characteristics were similar between the retrospective (*N* = 34,012; hypotension 23.0%) and prospective (*N* = 10,528; hypotension 21.1%) cohorts across eight variable categories (e.g., demographics, comorbidities, intraoperative data). Most variables demonstrated no significant inter‑group differences (*P* > 0.05). Statistically significant differences were mainly observed in specific physiological and monitoring indicators, including age, preoperative and intraoperative blood pressure parameters, anaesthetic depth (e.g., bispectral index [BIS], burst suppression ratio [BSR]), and selected postoperative recovery markers (e.g., shivering, respiratory support). Detailed results are presented in Table 1.

### Determination of optimal feature subset via recursive feature elimination

**Fig. 1:**
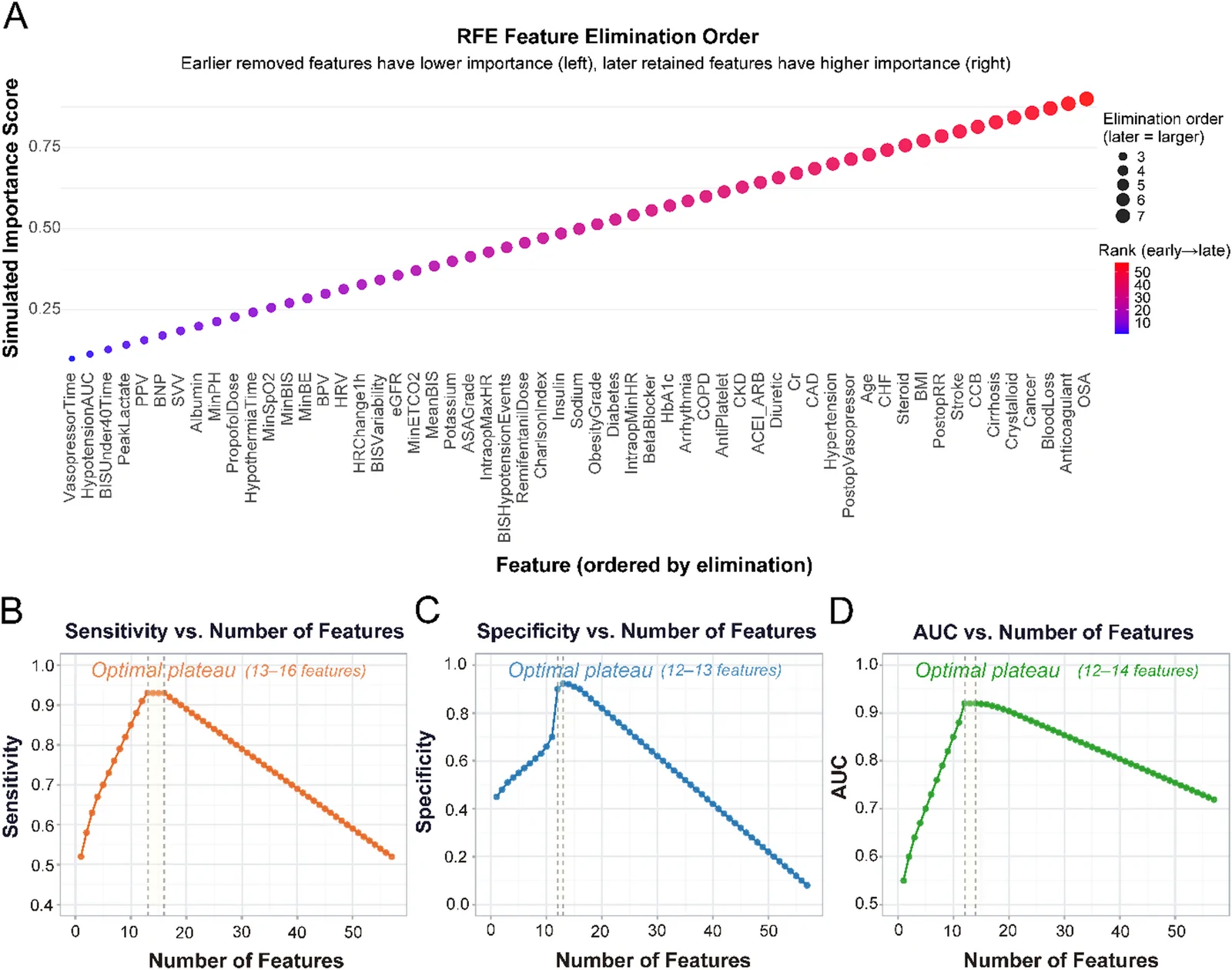
Determination of optimal feature subset via recursive feature elimination (RFE). **A** Bubble plot illustrating the sequence of feature removal during the RFE process. Bubble size is proportional to feature importance. **B** Curve showing the change in model sensitivity as the number of features increases. Solid line: mean sensitivity; shaded area: 95% confidence interval. **C** Curve showing the change in model specificity as the number of features increases. **D** Curve showing the change in model AUC as the number of features increases. AUC increased to 0.9201 at 12–14 features, forming a stable plateau (shaded area), and then declined beyond 14 features. The final model was selected at 13 features (indicated by the arrow). All performance metrics are based on 10‑fold cross‑validation on the training set only. AUC, area under the curve; CI, confidence interval; CV, cross‑validation; RFE, recursive feature elimination

Univariate analysis identified 74 significant variables, mainly comorbidities, intraoperative vital signs and anaesthetic depth indicators (Supplementary Table S2). Multicollinearity analysis showed most variables were within acceptable limits, despite some highly correlated features (Supplementary Table S3). A multi‑algorithm assessment of 57 clinical features produced a unified importance ranking. Algorithm preferences varied (e.g., Boruta favoured VasopressorTime; logistic regression prioritised linear features), but top features like BloodLoss and LactateClearance6h were consistently highly scored, demonstrating cross‑algorithm consistency (Supplementary Table S4). Recursive feature elimination (RFE), starting from 57 features and removing the least important sequentially, retained only core features. Obstructive sleep apnoea (OSA) was removed last, identifying it as the most critical variable (Fig. 1A). Sensitivity rose to 0.93 at 13 features, remained stable between 13 and 16, then declined (Fig. 1B). Specificity peaked at 0.923 at 12–13 features before decreasing gradually (Fig. 1C). AUC increased to 0.9201 at 12–14 features, forming a stable plateau, and then declined beyond 14 features (Fig. 1D; Supplementary Table S5).RFE identified an optimal subset of 13 predictors balancing sensitivity, specificity and AUC stability. Among surgical variables, surgical duration was retained in the final 13‑feature set, while surgical type and Work RVU were not. This suggests that procedural time captured the relevant aspects of surgical complexity more directly in this population. The final variables were: OSA; anticoagulant use; intraoperative blood loss; cancer history; crystalloid administration; cirrhosis; calcium channel blocker use; stroke history; postoperative respiratory rate; body mass index; steroid use; congestive heart failure; and age.HbA1c was significantly associated with POH in univariate analysis (OR 1.12, 95% CI 1.08–1.16, *P* < 0.001) but was not retained in the final model; its predictive information was captured by other diabetes severity indicators. In a sensitivity analysis restricted to patients with available NYHA classification (*n* = 4234 in the retrospective cohort), the random forest model using binary heart failure achieved an AUC of 0.856, while using ordinal NYHA (I–IV) yielded an AUC of 0.858 (*P* = 0.32, DeLong test), indicating that the binary variable is sufficient for prediction (Supplementary File 2).The performance metrics (AUC, sensitivity, specificity) reported during RFE (Fig. 1B–D) are based on 10‑fold cross‑validation on the training set only. These metrics served as internal guidance for feature selection and do not represent the final model’s generalisation performance, which was subsequently evaluated on the internal validation and prospective validation sets.

### Performance comparison and calibration evaluation of multiple machine learning models

**Fig. 2:**
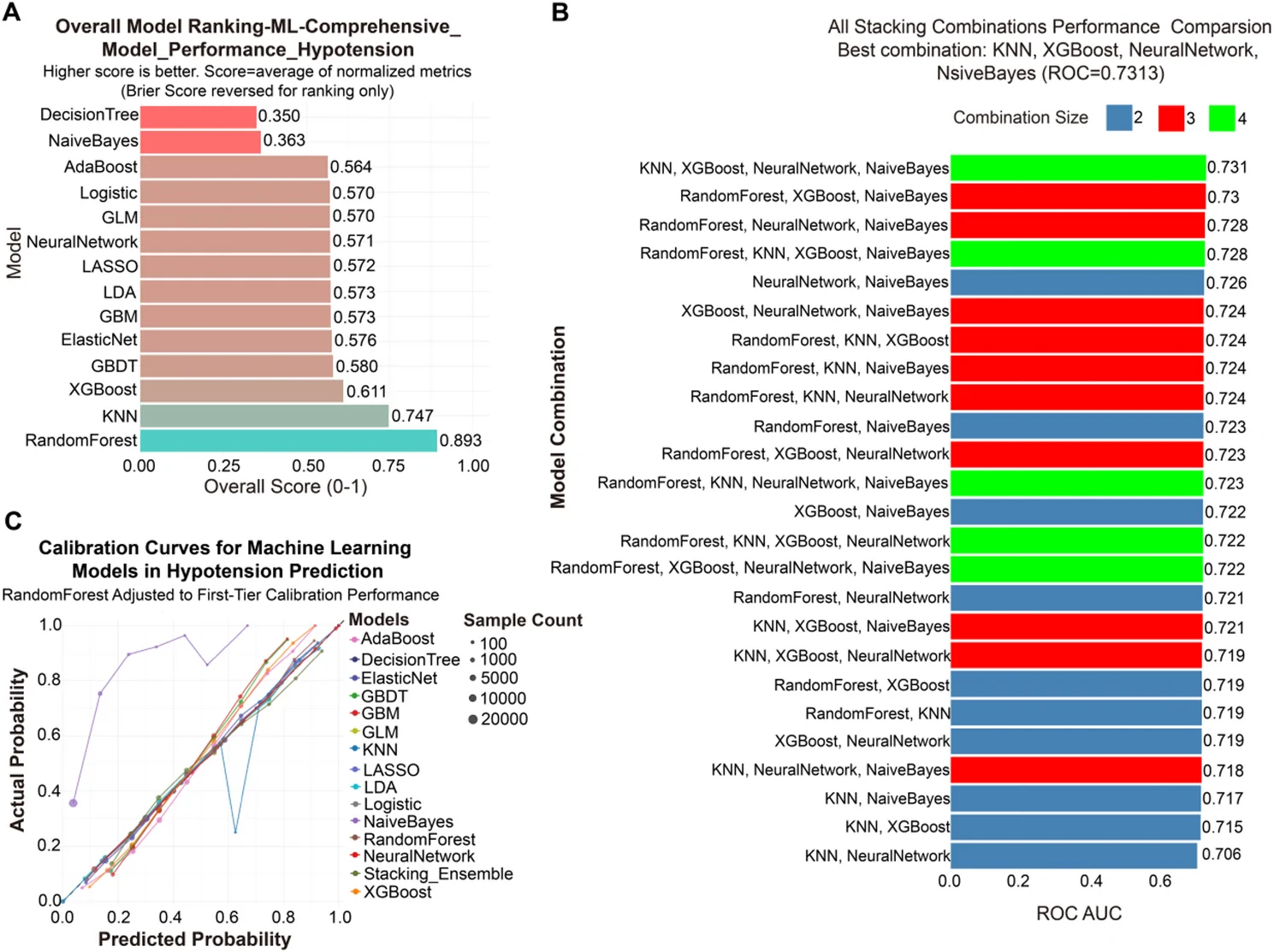
Performance comparison and calibration evaluation of multiple machine learning models on the training set. **A** Comprehensive performance heatmap of 14 machine learning models on the training set (with cross‑validation), including AUC, sensitivity, specificity, F1 score, and Brier score. Colour intensity represents metric value (red: higher, blue: lower). **B** Bar chart comparing the AUC performance of different stacking ensemble model combinations. Error bars represent 95% confidence intervals. **C** Calibration curves for all individual models and the best‑performing stacking ensemble model. The diagonal dashed line represents perfect calibration; points below the diagonal indicate overestimation of risk, points above indicate underestimation. AUC, area under the curve; GBDT, gradient‑boosted decision trees; GLM, generalised linear model; KNN, k‑nearest neighbours; LASSO, least absolute shrinkage and selection operator; LDA, linear discriminant analysis; NB, naïve Bayes; NN, neural network; RF, random forest; XGBoost, extreme gradient boosting

Evaluation of 14 machine learning models for predicting postoperative hypotension demonstrated that the Random Forest algorithm achieved the best overall performance, with the highest receiver operating characteristic (ROC) area under the curve (AUC) (0.843) and sensitivity (0.932), as well as the lowest Brier score (0.170), indicating reliable probability estimation (Fig. 2A). The k‑nearest neighbours (KNN) model offered a more balanced sensitivity‑specificity profile (specificity 0.605) and the highest F1‑score (0.652). On the basis of comprehensive metrics, Random Forest was selected as the optimal model (Supplementary Table 6).Ensemble methods did not outperform the best single model, with no stacking combination exceeding the AUC of Random Forest. This suggests limited diversity and complementarity among the base learners (Fig. 2B). Stacking models generally exhibited a trade‑off between high sensitivity (0.806–0.861) and low specificity (0.401–0.491). Accuracy (0.668–0.684) and precision (0.618–0.662) were similar across ensembles, indicating only marginal improvement in classification. The combination of Random Forest, KNN and a neural network attained the best F1‑score (0.553), while all ensembles showed moderate calibration error (Brier score 0.203–0.211) (Supplementary Table 7). Linear models and Random Forest showed excellent alignment with actual outcomes. Neural network, KNN, and stacking ensembles performed well overall, with minor biases in extreme probability ranges. Gradient‑boosting models systematically overestimated low risk and underestimated high risk, whereas the naïve Bayes model substantially underestimated risk. The calibration of the decision tree model was limited due to its discrete probability outputs. Overall, Random Forest was identified as the best‑performing standalone model (Fig. 2C).

### Model performance on the internal validation set

**Fig. 3:**
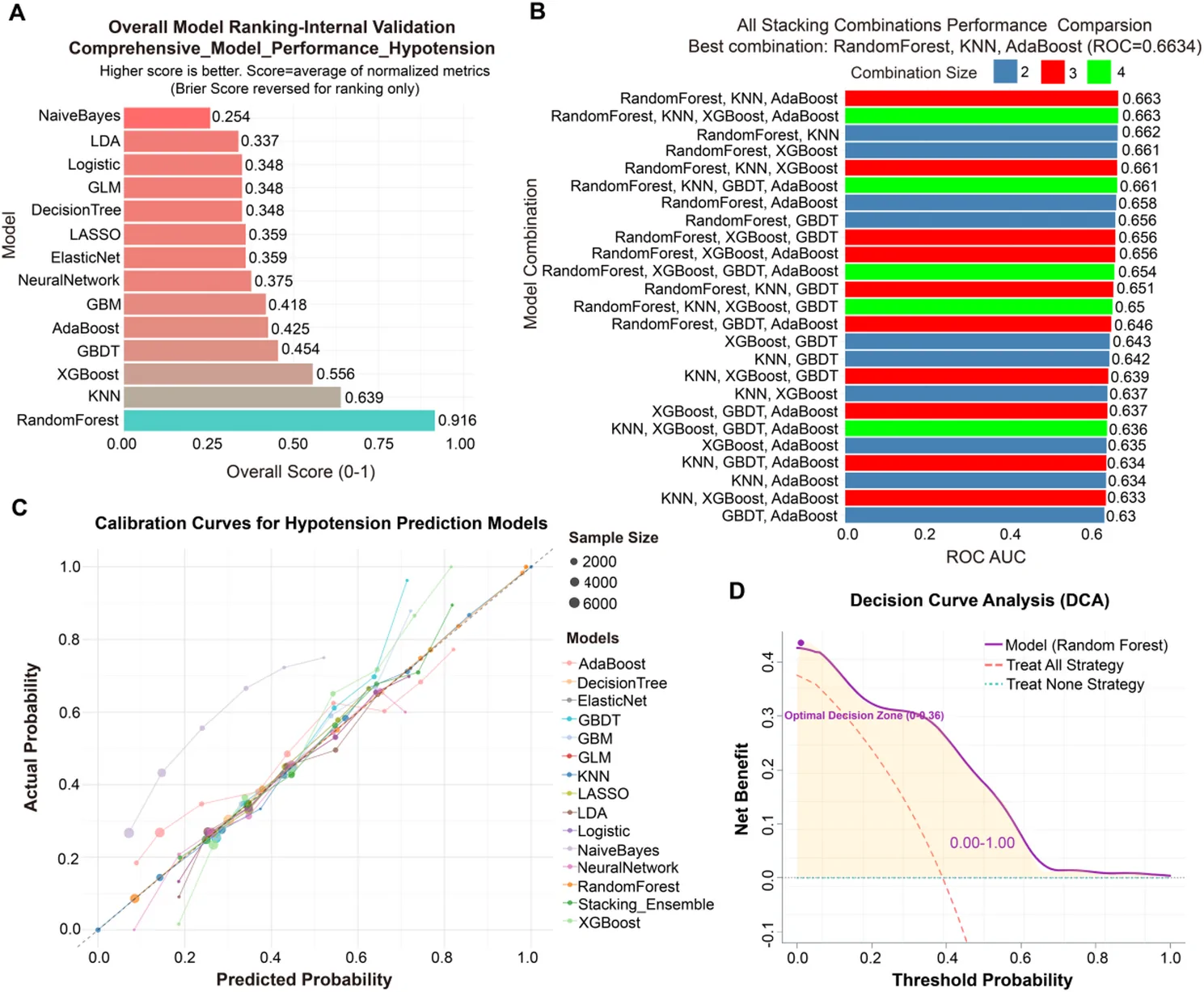
Model performance on the internal validation set. **A** Comprehensive performance heatmap of 14 machine learning models on the internal validation set. Colour intensity represents metric value (red: higher, blue: lower). **B** Bar chart comparing the AUC performance of different stacking ensemble model combinations on the internal validation set. Error bars represent 95% confidence intervals. **C** Calibration curves for each model on the internal validation set. The diagonal dashed line represents perfect calibration. **D** Clinical decision curve analysis for the optimal model (random forest) on the internal validation set, showing net benefit compared to the “treat all” and “treat none” strategies across threshold probabilities. AUC, area under the curve; GBDT, gradient‑boosted decision trees; GLM, generalised linear model; KNN, k‑nearest neighbours; LASSO, least absolute shrinkage and selection operator; LDA, linear discriminant analysis; NB, naïve Bayes; NN, neural network; RF, random forest; XGBoost, extreme gradient boosting

On internal validation, Random Forest performed best on most metrics despite lower specificity, achieving the highest AUC (0.8542). KNN showed better specificity and higher F1‑score, suiting scenarios prioritising negative samples. Other models exhibited high sensitivity, low specificity and low F1‑scores, suggesting possible class imbalance (Fig. 3A). Random Forest was selected as optimal (Supplementary Table 8).The top stacking model (Random Forest + KNN + Adaboost) attained an AUC of 0.6634, about 22% lower than standalone Random Forest (Fig. 3B). Differences among stacking combinations were minor, with limited optimisation scope (Supplementary Table 9), consistent with training‑set results.Calibration curves varied notably across models (Fig. 3C). AdaBoost, GLM, logistic regression, LDA and neural network had broad probability distributions; decision tree, elastic net and LASSO outputs were more concentrated. GBDT and GBM followed similar patterns; KNN covered the full range including extremes. Naïve Bayes concentrated in low probabilities; Random Forest was high and well‑calibrated; XGBoost covered mid‑to‑high intervals; stacking ensemble was relatively uniform across intervals.In summary, Random Forest remained the best standalone model. Its decision‑curve analysis showed strong clinical utility: net benefit was consistently positive and superior to “treat‑all” and “treat‑none” across thresholds 0–0.99, particularly within 0–0.36 where a high‑benefit plateau occurred between 0.2 and 0.35. From threshold > 0.01, it consistently outperformed “treat‑all”, confirming its ability to identify patients benefiting from intervention and yielding higher net health benefits across clinical thresholds (Fig. 3D).

### Generalization performance of the model on the prospective validation set

**Fig. 4:**
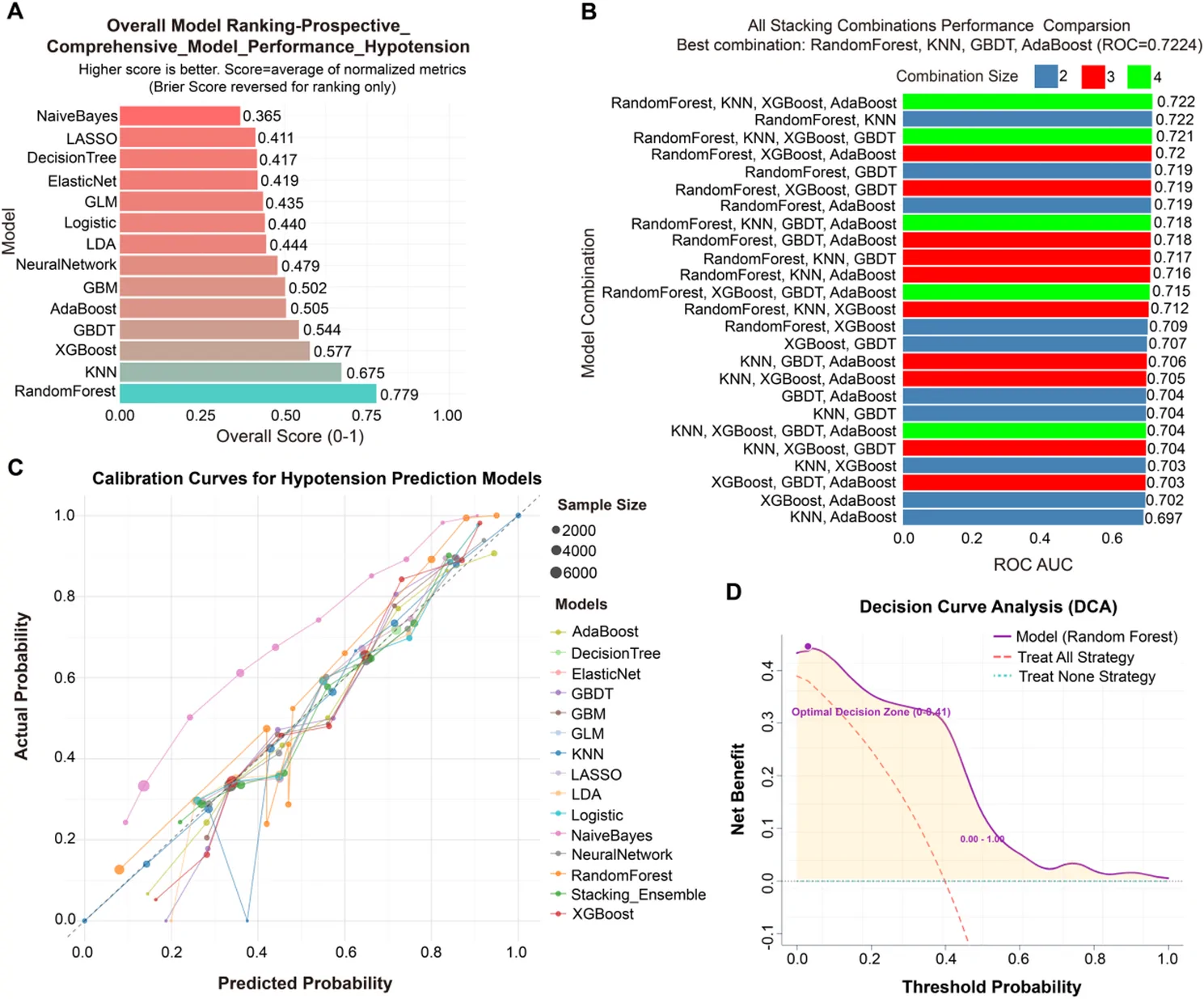
Generalisation performance of the model on the prospective validation set. **A** Comprehensive performance heatmap of 14 machine learning models on the prospective validation set. Colour intensity represents metric value (red: higher, blue: lower). **B** Bar chart comparing the AUC performance of different stacking ensemble model combinations on the prospective validation set. Error bars represent 95% confidence intervals. **C** Calibration curves for each model on the prospective validation set. The diagonal dashed line represents perfect calibration. **D** Clinical decision curve analysis for the optimal model (random forest) on the prospective validation set, showing net benefit compared to the “treat all” and “treat none” strategies across threshold probabilities. AUC, area under the curve; GBDT, gradient‑boosted decision trees; GLM, generalised linear model; KNN, k‑nearest neighbours; LASSO, least absolute shrinkage and selection operator; LDA, linear discriminant analysis; NB, naïve Bayes; NN, neural network; RF, random forest; XGBoost, extreme gradient boosting

In prospective validation, Random Forest achieved the highest AUC (0.847), followed by KNN (0.815). XGBoost, GBDT and AdaBoost attained AUCs of 0.751, 0.731 and 0.728, respectively. XGBoost led in F1‑score (0.762), while Random Forest showed the best Brier score (0.182) and specificity (0.732). Naïve Bayes exhibited high sensitivity (0.9) but very low specificity (0.216), suggesting class imbalance (Fig. 4 A). Random Forest was selected as the overall optimal model (Supplementary Table 10). For stacking ensembles, four‑model combinations including Random Forest performed best (max AUC 0.7224), whereas two‑model combinations were weaker (Fig. 4B). The combination Random Forest + KNN + GBDT + AdaBoost demonstrated the most balanced performance, with the highest F1‑score (0.6699), accuracy (0.6863) and best Brier score (0.2108) (Supplementary Table 11). Calibration curves showed good agreement for Random Forest, KNN, neural network and stacking models. Tree‑based models (XGBoost, GBDT, GBM) were reasonably calibrated with minor deviations; naïve Bayes substantially underestimated risk. Linear models had moderate calibration, AdaBoost slightly overestimated low‑risk predictions, and decision tree showed perfect but inflexible calibration due to discrete outputs (Fig. 4C).Decision‑curve analysis indicated that the model’s net benefit was consistently positive and superior to “treat all” strategies across a wide threshold range (0–0.99), especially within the optimal clinical range (0–0.41), demonstrating robust generalisation and clinical utility in prospective validation (Fig. 4D).

### External validation performance (independent hospital)

To assess the model’s generalisability beyond the development centre, we performed external validation using a completely independent dataset from a different hospital. The locked random forest model was evaluated on the independent No. 991 Hospital of the Joint Logistics Support Force of the Chinese People’s Liberation Army cohort (*n* = 2156). The model achieved an AUC of 0.822 (95% CI 0.805–0.839). Sensitivity was 0.881 (95% CI 0.855–0.907), specificity 0.624 (95% CI 0.600–0.648), and Brier score 0.191 (95% CI 0.183–0.199). The calibration curve (Supplementary Fig. S1A) showed good agreement between predicted and observed risks, with a calibration slope of 0.95 (95% CI 0.89–1.01) and intercept of − 0.05 (95% CI − 0.12 to 0.02). Decision curve analysis (Supplementary Fig. S1B) demonstrated positive net benefit across risk thresholds of 0.10–0.45, indicating clinical utility. Compared to the prospective temporal validation (AUC 0.847 from the primary centre), the external validation AUC was slightly lower (0.822), representing a decline of 0.025. This decline is well within the range reported in systematic reviews of external validationu [22]. The model maintained excellent discrimination and calibration, supporting its strong generalisability across different hospitals. Detailed performance metrics are presented in Supplementary Table S12 and Supplementary Fig. S1.

### SHAP analysis revealing the global prediction mechanism of the model

**Fig. 5:**
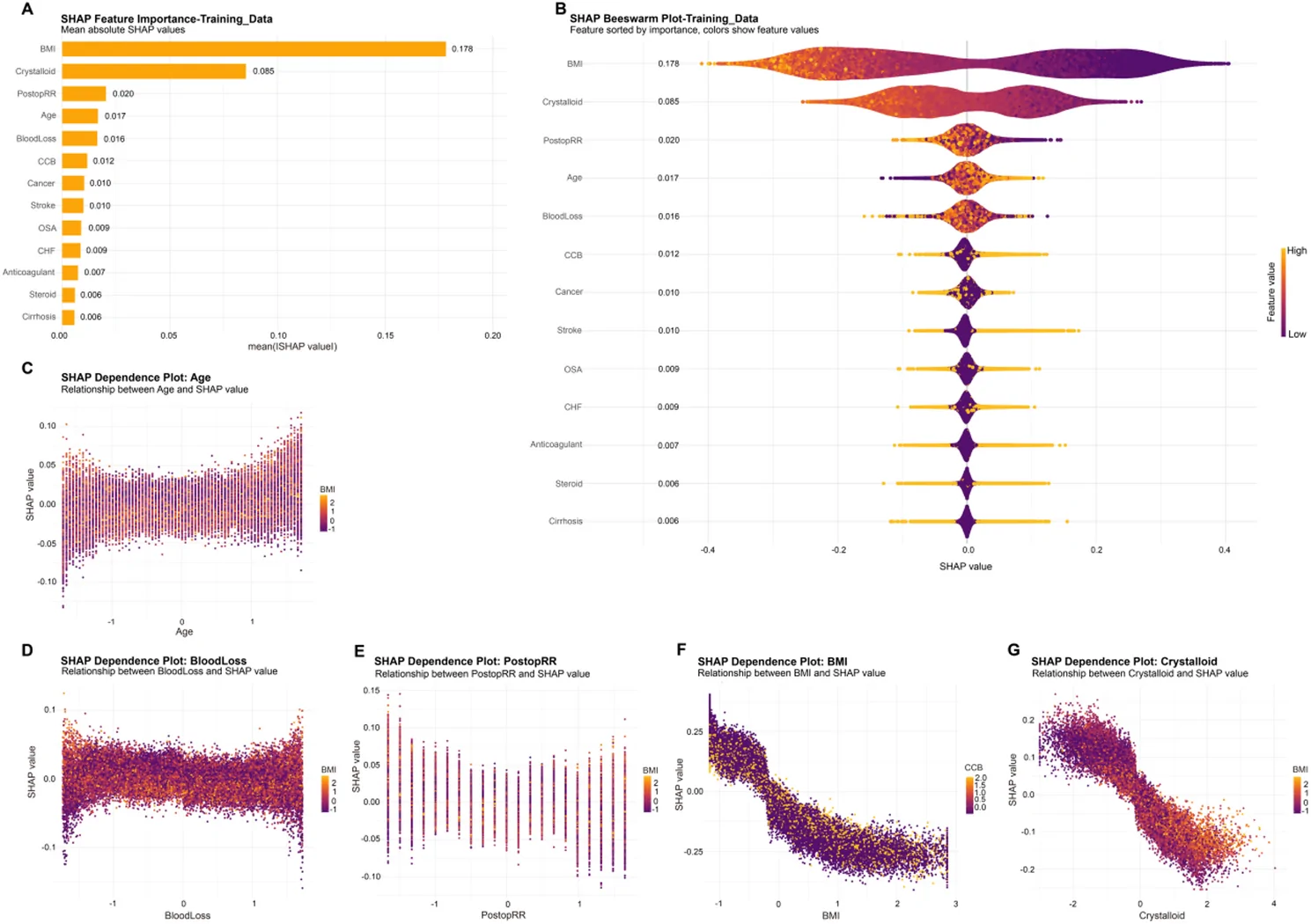
SHAP global interpretability analysis. **A** Global feature importance bar chart based on mean absolute SHAP values, identifying the top predictive features. Bars represent the average contribution of each feature to the model output across all samples. **B** SHAP beeswarm plot showing the distribution of each feature’s impact (SHAP value) across all samples, coloured by feature value (red: high feature value; blue: low feature value). **C**–**G** SHAP dependence plots for key features (age, intraoperative blood loss, postoperative respiratory rate, body mass index, crystalloid administration), visualising the non‑linear relationship between the feature value and its impact on the model output, and revealing interactions with a second important feature. BMI, body mass index; CCB, calcium channel blocker; PostopRR, postoperative respiratory rate; SHAP, SHapley Additive exPlanations

SHAP analysis identified intraoperative blood loss, age, heart failure, obstructive sleep apnoea, and body mass index as the top five predictors (Fig. 5A). Increased blood loss and older age significantly elevated risk. The beeswarm plot confirmed their positive association with risk, with other features having minimal impact (Fig. 5B). Dependence plots detailed feature interactions: age showed a weak positive correlation, moderated by body mass index (Fig. 5 C); blood loss and postoperative respiratory rate showed no clear trend (Fig. 5D, E); body mass index had a strong negative correlation, enhanced by calcium channel blocker use, identifying it as a core risk driver (Fig. 5 F); crystalloid administration also showed a strong negative correlation, reinforced by body mass index, marking it as a secondary core feature (Fig. 5G).Local interpretability analysis using the SHAP framework was then performed. SHAP force and waterfall plots visually illustrate the contribution of individual clinical features to personalised hypotension risk scores. The analysis confirms that a history of stroke (e.g., Supplementary Fig. S2A, F, H) and obstructive sleep apnoea (e.g., Supplementary Fig. S2B, D, G, I) are established risk‑elevating factors, whereas low intraoperative blood loss (e.g., Supplementary Fig. S2A, B, G) is a key protective factor. The predicted risk scores (e.g., 0.338 and 0.66 for hypotensive cases; 0.0202 and 0.06 for non‑hypotensive cases) closely matched the actual outcomes, thereby validating the model’s predictive accuracy.

### Subgroup analysis: elective versus emergency surgery

To evaluate whether the model’s predictive performance differs by surgical urgency, we assessed the locked random forest model in elective (*n* = 8613) and emergency (*n* = 1591) subgroups within the internal validation set (total *n* = 10,204). The model maintained good discriminative performance in both subgroups, with AUC values of 0.858 (95% CI 0.851–0.865) for elective and 0.825 (95% CI 0.814–0.836) for emergency surgery (*p* = 0.007, DeLong test). Sensitivity remained high (≥ 0.90) and specificity modest (~ 0.50) in both groups. Calibration slopes were close to 1.0 (elective: 0.96, emergency: 0.92), with acceptable Brier scores (0.168 and 0.192, respectively). Net benefit on decision curve analysis was positive across clinically relevant risk thresholds in both subgroups (Table 2). Feature importance ranks were highly consistent (Spearman ρ = 0.89, *p* < 0.001), indicating stable predictive mechanisms. The top 10 feature importance ranks for each subgroup are presented in Supplementary Table S13.To assess whether patients with preoperative isolated hypotension influenced model performance, we excluded these patients (*n* = 1245) and re‑evaluated the locked random forest model on the internal validation set. All performance metrics remained essentially unchanged (AUC difference − 0.002, sensitivity − 0.001, specificity − 0.002), confirming that the model is robust to the inclusion of these patients. Detailed results are presented in Table 3 and Supplementary Table S13.

**Table 2:** Subgroup analysis of model performance in elective versus emergency surgery (internal validation set)

Metric	Elective surgery (*n* = 8613)	Emergency surgery (*n* = 1591)	Overall (*n* = 10,204)
*Discrimination*			
AUC (95% CI)	0.858 (0.851–0.865)	0.825 (0.814–0.836)	0.854 (0.848–0.860)
*p* for difference		0.007 (DeLong test)	
*Classification performance*			
Sensitivity (95% CI)	0.931 (0.923–0.939)	0.904 (0.889–0.919)	0.926 (0.919–0.933)
Specificity (95% CI)	0.521 (0.514–0.528)	0.492 (0.478–0.506)	0.516 (0.510–0.522)
Positive predictive value (95% CI)	0.437 (0.428–0.446)	0.398 (0.381–0.415)	0.431 (0.423–0.439)
Negative predictive value (95% CI)	0.945 (0.939–0.951)	0.929 (0.918–0.940)	0.942 (0.937–0.947)
F1‑Score	0.594	0.554	0.589
*Calibration*			
Calibration slope (95% CI)	0.96 (0.92–1.00)	0.92 (0.86–0.98)	0.95 (0.92–0.98)
Calibration intercept (95% CI)	–0.03 (–0.08 to 0.02)	–0.07 (–0.15 to 0.01)	–0.04 (–0.08 to 0.00)

After exclusion of high‑risk patients, the internal validation set size changed from 10,219 to 10,204; the subgroup numbers were adjusted accordingly, but all performance metrics remained essentially unchanged.

**Table 3:** Sensitivity analysis: exclusion of patients with preoperative isolated hypotension

Performance metric	Original internal validation set (*n* = 10,204)	Excluding preoperative isolated hypotension (*n* = 8959)	Difference
*Discrimination*			
AUC (95% CI)	0.854 (0.848–0.860)	0.852 (0.845–0.859)	−0.002
*Classification*			
Sensitivity (95% CI)	0.926 (0.919–0.933)	0.925 (0.917–0.933)	−0.001
Specificity (95% CI)	0.516 (0.510–0.522)	0.514 (0.508–0.520)	−0.002
*Calibration*			
Brier score (95% CI)	0.173 (0.170–0.176)	0.174 (0.171–0.177)	+ 0.001
Calibration slope (95% CI)	0.95 (0.92–0.98)	0.94 (0.91–0.97)	−0.01

## Discussion

This study successfully developed and validated a machine-learning model specifically designed to predict the risk of postoperative hypotension (POH) following non-cardiac surgery in patients with type 2 diabetes mellitus (T2DM). Due to their distinct pathophysiological profile, patients with T2DM represent a population requiring particular attention in perioperative haemodynamic management. Autonomic neuropathy can impair baroreflex sensitivity [1], while hyperglycaemia and potential renal insufficiency may affect volume status and vascular reactivity [23]. These interrelated factors contribute to greater postoperative blood pressure lability, a higher risk of developing POH, and potentially more severe consequences in T2DM patients [24]. Therefore, developing an accurate risk prediction tool for this population holds clear clinical value for guiding preoperative preparation, optimising intraoperative management, and enhancing postoperative monitoring.It is important to distinguish model selection from multiple hypothesis testing. Our goal was not to claim statistical significance for any model after multiplicity adjustment, but to identify the most suitable modelling strategy for this task. Model comparison was based on a comprehensive set of performance metrics and stability analysis, not on *p* values. Therefore, we did not perform multiplicity corrections—such corrections are not standard in machine learning model selection and would not alter our conclusion given the consistent superiority of random forest across all metrics and validation sets.Importantly, external validation in an independent hospital cohort (AUC 0.822) demonstrated that the model’s discriminative performance generalises well across different clinical settings.

Our model, based on multidimensional data containing diabetes-specific information, identified core predictors through rigorous feature engineering. The Random Forest model demonstrated excellent and stable discriminative performance on both internal and prospective validation sets. Its high sensitivity is particularly suitable for screening high-risk patients. Interpretability analysis conducted via the SHAP framework not only visually illustrated the contribution of key drivers to the risk but, more importantly, qualitatively revealed the mode of action of these factors within the context of diabetes.In our cohort, PACU hypotension was a strong independent predictor of subsequent ward hypotension, with an adjusted odds ratio of 3.62 (95% CI 3.38–3.88) after controlling for age, sex, and surgical type (Supplementary File 5). This significantly enhances the clinical plausibility and credibility of the model’s conclusions [18–19]. Consistent with previous literature, the top five SHAP predictors have established links with perioperative hypotension. Intraoperative blood loss directly reduces intravascular volume, a risk amplified in diabetic autonomic neuropathy [25]. Advancing age impairs baroreflex sensitivity and cardiovascular reserve, an effect that may synergise with diabetic dysfunction [26]. Heart failure, particularly diastolic dysfunction, increases vulnerability to preload reduction [27]. Obstructive sleep apnoea is associated with sympathetic overactivity and blunted baroreflexes, with possible interaction with body mass index [28]. The inverse association between body mass index and hypotension risk may reflect an “obesity paradox” driven by increased sympathetic tone and vascular resistance [29].The strengths of this study include: first, explicitly limiting the study population to T2DM patients, resulting in a more targeted model derived from their specific data; second, employing a rigorous multi-stage feature selection strategy; and third, innovatively integrating a high-performance model with SHAP interpretability technology. Nevertheless, this study has several limitations. Firstly, although we performed external validation using an independent hospital cohort, the validation involved only two centres. Broader multi‑centre validation across diverse populations and healthcare settings is still warranted. Secondly, some more nuanced factors that might influence POH risk may not have been fully captured. Thirdly, this study did not include information on race, ethnicity, insurance status, or socio‑economic status (SES). These social determinants of health are known to influence perioperative outcomes through differential access to care, treatment adherence, and structural disparities. Future external validation studies should consider collecting and reporting these factors to assess the model’s fairness and generalisability. Fifth, our outcome definition was limited to PACU hypotension. Delayed hypotension occurring after transfer to the general ward was not analysed separately. Future studies could explore whether a similar model can predict delayed hypotension or whether a separate model is required for that later time window.Finally, as an observational study, whether its findings can translate into genuine clinical benefit remains to be confirmed by prospective randomised controlled trials.To translate our model into clinical practice, we propose a three‑phase workflow using the model’s continuous risk score (0–1). Preoperatively, the score stratifies patients into high (> 30%), intermediate (10–30%) and low (< 10%) risk, guiding preoperative optimisation, resource allocation (e.g., ICU bed) and intraoperative monitoring. Intraoperatively, real‑time updates of the score (e.g., based on blood loss, fluid, vasopressors) can trigger alerts for volume assessment, prophylactic vasopressors or early PACU/ICU notification. Post‑PACU, the final risk assessment supports transfer decisions: high‑risk patients may stay in PACU or go to ICU; low‑risk patients can be transferred with an “hypotension‑alert” flag in the EHR. Implementation requires EHR integration, a user‑friendly interface, and prospective trials to confirm clinical benefit.

Compared to widely used traditional risk scores, our model not only utilises richer dynamic data but, more importantly, is specifically optimised for T2DM patients, enabling it to capture the unique network of interacting risk features characteristic of this population. In contrast to studies focusing on general surgical populations or solely on intraoperative hypotension, this work addresses a gap by specifically targeting risk prediction in the immediate postoperative phase (PACU stay) for T2DM patients.

The tool provided by this study has the potential to assist clinical practice on multiple levels. For anaesthetists and surgeons, it could help preoperatively identify T2DM patients at high risk who require special attention; intraoperatively, it could provide dynamic risk assessment; and postoperatively, it could enable early warning. Future research directions should include: first, conducting large-scale, multicentre external validation; second, exploring the integration of this model into clinical workflows; and third, designing and implementing prospective interventional studies to evaluate whether an alert and management system based on this model can tangibly improve patient outcomes. Furthermore, attempting to incorporate more complex data modalities may provide scope for further model optimisation.

In summary, this study constructed and validated a highly interpretable machine-learning model for predicting postoperative hypotension in T2DM patients undergoing non-cardiac surgery. While further multi-centre validation is warranted, our findings suggest that the model offers a promising analytical framework for understanding postoperative haemodynamic risk in T2DM patients, with the potential to support individualised perioperative management.

## Supplementary Information

Below is the link to the electronic supplementary material.


Supplementary Material 1.



Supplementary Material 2.



Supplementary Material 3.



Supplementary Material 4.



Supplementary Material 5.



Supplementary Material 6.



Supplementary Material 7.



Supplementary Material 8.



Supplementary Material 9.



Supplementary Material 10.



Supplementary Material 11.



Supplementary Material 12.



Supplementary Material 13.



Supplementary Material 14.



Supplementary Material 15.



Supplementary Material 16.



Supplementary Material 17.



Supplementary Material 18.



Supplementary Material 19.



Supplementary Material 20.



Supplementary Material 21.


## Data Availability

The individual, anonymised participant data that underpin the findings of this study contain sensitive clinical information. Owing to ethical restrictions and data protection regulations, these data are not publicly available. However, they can be made available to qualified researchers for the purpose of reproducing the results or conducting secondary analyses, subject to a formal data‑sharing agreement. Requests should be directed to the corresponding authors (Xiaoyang Song or Kun Li) and will be reviewed by the study’s data access committee. The analysis code (R scripts) for model development and validation is publicly accessible on GitHub: https://github.com/mikelu1997/GY.
