# Supplementary material for: An interpretable machine learning model for predicting postoperative hypotension in type 2 diabetes mellitus undergoing non‑cardiac surgery
Source: Cardiovasc Diabetol. 2026 Jun 22;25:265. doi: 10.1186/s12933-026-03256-3 (PMC13536729; doi:10.1186/s12933-026-03256-3)
Supplement: Supplementary file 3 [file 12933_2026_3256_MOESM11_ESM.docx]

Supplementary Table S10. Comprehensive performance metrics of the stacking ensemble combinations on the prospective validation set with 95% confidence intervals for AUC.

| Model | AUC | 95% CI for AUC | Sensitivity | Specificity | Accuracy | Precision | F1‑Score | Brier Score | TrainingTime | Combination Size | Combination |
| --- | --- | --- | --- | --- | --- | --- | --- | --- | --- | --- | --- |
| Stacking_Ensemble | 0.7220 | 0.713–0.731 | 0.7140 | 0.6412 | 0.6781 | 0.6852 | 0.6625 | 0.2115 | NA | 2 | RandomForest, KNN |
| Stacking_Ensemble | 0.7089 | 0.699–0.719 | 0.7018 | 0.6347 | 0.6687 | 0.6739 | 0.6537 | 0.2155 | NA | 2 | RandomForest, XGBoost |
| Stacking_Ensemble | 0.7191 | 0.710–0.728 | 0.7200 | 0.6350 | 0.6781 | 0.6877 | 0.6603 | 0.2121 | NA | 2 | RandomForest, GBDT |
| Stacking_Ensemble | 0.7187 | 0.709–0.728 | 0.7179 | 0.6410 | 0.6800 | 0.6881 | 0.6637 | 0.2121 | NA | 2 | RandomForest, AdaBoost |
| Stacking_Ensemble | 0.7030 | 0.693–0.713 | 0.7213 | 0.6034 | 0.6632 | 0.6776 | 0.6384 | 0.2172 | NA | 2 | KNN, XGBoost |
| Stacking_Ensemble | 0.7039 | 0.694–0.714 | 0.6990 | 0.6367 | 0.6683 | 0.6726 | 0.6541 | 0.2170 | NA | 2 | KNN, GBDT |
| Stacking_Ensemble | 0.6968 | 0.687–0.707 | 0.7065 | 0.6049 | 0.6565 | 0.6668 | 0.6343 | 0.2200 | NA | 2 | KNN, AdaBoost |
| Stacking_Ensemble | 0.7070 | 0.697–0.717 | 0.7312 | 0.6006 | 0.6668 | 0.6845 | 0.6398 | 0.2160 | NA | 2 | XGBoost, GBDT |
| Stacking_Ensemble | 0.7022 | 0.692–0.712 | 0.7227 | 0.6001 | 0.6623 | 0.6776 | 0.6365 | 0.2175 | NA | 2 | XGBoost, AdaBoost |
| Stacking_Ensemble | 0.7043 | 0.694–0.714 | 0.7182 | 0.6205 | 0.6700 | 0.6813 | 0.6495 | 0.2168 | NA | 2 | GBDT, AdaBoost |
| Stacking_Ensemble | 0.7116 | 0.702–0.721 | 0.7161 | 0.6312 | 0.6742 | 0.6834 | 0.6562 | 0.2147 | NA | 3 | RandomForest, KNN, XGBoost |
| Stacking_Ensemble | 0.7168 | 0.707–0.727 | 0.7302 | 0.6300 | 0.6808 | 0.6939 | 0.6604 | 0.2124 | NA | 3 | RandomForest, KNN, GBDT |
| Stacking_Ensemble | 0.7163 | 0.707–0.726 | 0.7239 | 0.6327 | 0.6789 | 0.6899 | 0.6600 | 0.2131 | NA | 3 | RandomForest, KNN, AdaBoost |
| Stacking_Ensemble | 0.7188 | 0.709–0.729 | 0.7154 | 0.6327 | 0.6747 | 0.6834 | 0.6571 | 0.2126 | NA | 3 | RandomForest, XGBoost, GBDT |
| Stacking_Ensemble | 0.7202 | 0.711–0.730 | 0.7162 | 0.6312 | 0.6743 | 0.6835 | 0.6563 | 0.2119 | NA | 3 | RandomForest, XGBoost, AdaBoost |
| Stacking_Ensemble | 0.7177 | 0.708–0.727 | 0.7114 | 0.6455 | 0.6789 | 0.6847 | 0.6645 | 0.2127 | NA | 3 | RandomForest, GBDT, AdaBoost |
| Stacking_Ensemble | 0.7037 | 0.694–0.714 | 0.7234 | 0.6076 | 0.6663 | 0.6808 | 0.6421 | 0.2168 | NA | 3 | KNN, XGBoost, GBDT |
| Stacking_Ensemble | 0.7048 | 0.695–0.715 | 0.7300 | 0.6017 | 0.6668 | 0.6840 | 0.6402 | 0.2167 | NA | 3 | KNN, XGBoost, AdaBoost |
| Stacking_Ensemble | 0.7063 | 0.697–0.716 | 0.7248 | 0.6101 | 0.6683 | 0.6828 | 0.6444 | 0.2162 | NA | 3 | KNN, GBDT, AdaBoost |
| Stacking_Ensemble | 0.7027 | 0.693–0.713 | 0.7287 | 0.6036 | 0.6671 | 0.6836 | 0.6411 | 0.2167 | NA | 3 | XGBoost, GBDT, AdaBoost |
| Stacking_Ensemble | 0.7214 | 0.712–0.731 | 0.7114 | 0.6470 | 0.6797 | 0.6852 | 0.6656 | 0.2117 | NA | 4 | RandomForest, KNN, XGBoost, GBDT |
| Stacking_Ensemble | 0.7178 | 0.708–0.728 | 0.7177 | 0.6399 | 0.6794 | 0.6876 | 0.6629 | 0.2126 | NA | 4 | RandomForest, KNN, XGBoost, AdaBoost |
| Stacking_Ensemble | 0.7224 | 0.713–0.732 | 0.7252 | 0.6462 | 0.6863 | 0.6954 | 0.6699 | 0.2108 | NA | 4 | RandomForest, KNN, GBDT, AdaBoost |
| Stacking_Ensemble | 0.7145 | 0.705–0.724 | 0.7183 | 0.6313 | 0.6755 | 0.6852 | 0.6572 | 0.2135 | NA | 4 | RandomForest, XGBoost, GBDT, AdaBoost |
| Stacking_Ensemble | 0.7037 | 0.694–0.714 | 0.7399 | 0.6014 | 0.6717 | 0.6919 | 0.6435 | 0.2166 | NA | 4 | KNN, XGBoost, GBDT, AdaBoost |
| Footnote: 95% confidence intervals for AUC were calculated using bootstrap resampling with 1,000 replicates. For each replicate, the prospective validation set was sampled with replacement, the stacking ensemble model was evaluated (without retraining), and AUC was computed. The 2.5th and 97.5th percentiles of the bootstrap distribution are reported. The CI widths are approximately ±0.009 to ±0.011, reflecting the sample size (n=10,528) and model stability. TrainingTime was not recorded (NA). | | | | | | | | | | | |
