# Supplementary material for: An interpretable machine learning model for predicting postoperative hypotension in type 2 diabetes mellitus undergoing non‑cardiac surgery
Source: Cardiovasc Diabetol. 2026 Jun 22;25:265. doi: 10.1186/s12933-026-03256-3 (PMC13536729; doi:10.1186/s12933-026-03256-3)
Supplement: Supplementary file 4 [file 12933_2026_3256_MOESM12_ESM.docx]

Table S12. Top 10 feature importance ranks in each subgroup.

| Rank | Elective Surgery | Emergency Surgery |
| --- | --- | --- |
| 1 | Intraoperative blood loss | Intraoperative blood loss |
| 2 | Age | Age |
| 3 | Heart failure | Heart failure |
| 4 | Obstructive sleep apnoea | Obstructive sleep apnoea |
| 5 | Body mass index | Body mass index |
| 6 | Anticoagulant use | Anticoagulant use |
| 7 | Crystalloid administration | Crystalloid administration |
| 8 | Cancer history | Cirrhosis |
| 9 | Cirrhosis | Cancer history |
| 10 | Calcium channel blocker use | Calcium channel blocker use |
| The rank correlation (Spearman P = 0.89) confirms that the model relies on the same predictors regardless of surgical urgency. | | |
