# Supplementary material for: An interpretable machine learning model for predicting postoperative hypotension in type 2 diabetes mellitus undergoing non‑cardiac surgery
Source: Cardiovasc Diabetol. 2026 Jun 22;25:265. doi: 10.1186/s12933-026-03256-3 (PMC13536729; doi:10.1186/s12933-026-03256-3)
Supplement: Supplementary file 5 [file 12933_2026_3256_MOESM13_ESM.docx]

Supplementary Table S13. Performance of the random forest model on the cross‑campus external validation cohort

| Metric | Value (95% CI) |
| --- | --- |
| AUC | 0.822 (0.805–0.839) |
| Sensitivity | 0.881 (0.855–0.907) |
| Specificity | 0.624 (0.600–0.648) |
| Positive predictive value | 0.432 (0.408–0.456) |
| Negative predictive value | 0.945 (0.931–0.959) |
| F1‑score | 0.580 |
| Brier score | 0.191 (0.183–0.199) |
| Calibration slope | 0.95 (0.89–1.01) |
| Calibration intercept | –0.05 (–0.12 to 0.02) |
