# Supplementary material for: An interpretable machine learning model for predicting postoperative hypotension in type 2 diabetes mellitus undergoing non‑cardiac surgery
Source: Cardiovasc Diabetol. 2026 Jun 22;25:265. doi: 10.1186/s12933-026-03256-3 (PMC13536729; doi:10.1186/s12933-026-03256-3)
Supplement: Supplementary file 6 [file 12933_2026_3256_MOESM14_ESM.docx]

1. Background

To support the clinical rationale for focusing on PACU hypotension (the primary outcome of our model), we analysed the association between PACU hypotension and subsequent ward hypotension (hypotension occurring after transfer to the general ward). Additionally, we compared the predictor profiles of the two outcomes to assess whether they are distinct entities.

2. Methods

2.1 Study Population

We used the retrospective cohort (N = 34,012) for this analysis. PACU hypotension was defined as any SBP <90 mmHg during PACU stay (same as the primary outcome). Ward hypotension was defined as any SBP <90 mmHg after transfer to the general ward within the first 48 hours post‑surgery.

2.2 Statistical Analysis

Association analysis: Logistic regression was used to estimate the odds ratio (OR) for ward hypotension comparing patients with vs. without PACU hypotension, adjusted for age, sex, and surgical type.

Predictor overlap analysis: We extracted the top 10 predictors (by SHAP importance) for the PACU hypotension model (as reported in the main manuscript) and compared them with the top 10 predictors for a separate random forest model developed to predict ward hypotension (using the same candidate variables but trained on the same retrospective cohort).

3. Results

3.1 Association Between PACU Hypotension and Ward Hypotension

| Exposure | Ward Hypotension (n, %) | OR (95% CI) | Adjusted OR* (95% CI) |
| --- | --- | --- | --- |
| No PACU hypotension (n=26,228) | 3,410 (13.0%) | Reference | Reference |
| PACU hypotension (n=7,834) | 3,845 (49.1%) | 6.54 (6.18–6.93) | 3.62 (3.38–3.88) |

Adjusted for age, sex, and surgical type.

Interpretation: After adjusting for confounders, patients with PACU hypotension had a 3.62‑fold higher odds of developing ward hypotension compared to those without PACU hypotension. This strong association supports the clinical relevance of predicting PACU hypotension as an early warning for later complications.

3.2 Predictor Overlap Between PACU and Ward Hypotension Models

| Rank | PACU Hypotension Model (Top 10 Predictors) | Ward Hypotension Model (Top 10 Predictors) |
| --- | --- | --- |
| 1 | Intraoperative blood loss | Intraoperative blood loss |
| 2 | Age | Age |
| 3 | Heart failure | Heart failure |
| 4 | Obstructive sleep apnoea | Surgical duration |
| 5 | Body mass index | Postoperative infection |
| 6 | Anticoagulant use | Crystalloid administration |
| 7 | Crystalloid administration | Body mass index |
| 8 | Cancer history | Anticoagulant use |
| 9 | Cirrhosis | Fever (post‑PACU) |
| 10 | Calcium channel blocker use | Obstructive sleep apnoea |

Overlap: 7 of the top 10 predictors are shared between the two models (blood loss, age, heart failure, BMI, anticoagulant use, crystalloid, OSA). However, the ward hypotension model uniquely includes surgical duration, postoperative infection, and fever, which are factors that may develop after PACU discharge.

Interpretation: The substantial overlap in predictors indicates that the two outcomes share common risk factors, but the ward hypotension model captures additional post‑operative complications. This justifies our focused approach on PACU hypotension while acknowledging that delayed hypotension may require separate modelling.

4. Conclusion

PACU hypotension is a strong independent predictor of ward hypotension (adjusted OR 3.62).

The two outcomes share most top predictors, but ward hypotension is additionally influenced by post‑PACU events (infection, fever, prolonged surgery).

These findings support the clinical utility of predicting PACU hypotension as an early warning tool, while also suggesting that a separate model for delayed hypotension might be developed in future research.
