# Supplementary material for: An interpretable machine learning model for predicting postoperative hypotension in type 2 diabetes mellitus undergoing non‑cardiac surgery
Source: Cardiovasc Diabetol. 2026 Jun 22;25:265. doi: 10.1186/s12933-026-03256-3 (PMC13536729; doi:10.1186/s12933-026-03256-3)
Supplement: Supplementary file 7 [file 12933_2026_3256_MOESM16_ESM.docx]

1. Background

To assess whether using a binary variable (presence/absence) for heart failure is adequate compared to the more detailed New York Heart Association (NYHA) functional classification, we performed a sensitivity analysis in the subset of patients with available NYHA data. This analysis addresses Reviewer 3’s concern regarding the quantification of heart failure severity.

2. NYHA Classification Availability

| Cohort | Total patients | Heart failure patients (n, %) | NYHA data available (n, % of HF) |
| --- | --- | --- | --- |
| Retrospective | 34,012 | 7,642 (22.5%) | 4,234 (55.4%) |
| Prospective | 10,528 | 2,242 (21.3%) | 1,456 (64.9%) |

3. Distribution of NYHA Classes (Retrospective Cohort)

| NYHA class | n | % |
| --- | --- | --- |
| I | 1,245 | 29.4% |
| II | 2,034 | 48.1% |
| III | 789 | 18.6% |
| IV | 166 | 3.9% |

4. Univariate Association of NYHA Class with POH (Internal Validation Set)

| NYHA class | OR (95% CI) | P value |
| --- | --- | --- |
| I (reference) | 1.00 | – |
| II | 1.42 (1.28–1.58) | <0.001 |
| III | 1.68 (1.52–1.86) | <0.001 |
| IV | 2.15 (1.92–2.41) | <0.001 |
| Per class increase | 1.25 (1.18–1.32) | <0.001 |
| Interpretation: Higher NYHA class was associated with a stepwise increase in POH risk, confirming the clinical relevance of heart failure severity. | | |

5. Model Performance Comparison (Internal Validation Set)

| Heart failure variable | AUC (95% CI) | Sensitivity | Specificity |
| --- | --- | --- | --- |
| Binary (presence/absence) | 0.856 (0.848–0.864) | 0.928 | 0.518 |
| Ordinal NYHA (I–IV) | 0.858 (0.850–0.866) | 0.931 | 0.521 |
| Difference | –0.002 | –0.003 | –0.003 |
| DeLong test for AUC difference: P = 0.32 | | | |

6. Interpretation

The AUC difference between the binary and ordinal NYHA models was negligible (−0.002) and not statistically significant (P = 0.32). The binary heart failure variable therefore captures the predictive information adequately for the main analysis. The ordinal NYHA model did not provide a meaningful improvement, supporting the use of the simpler binary variable given incomplete NYHA documentation in the full cohort.

7. Conclusion

NYHA classification shows a clear dose‑response relationship with POH risk.

However, replacing NYHA with a binary heart failure variable does not materially affect model performance (ΔAUC = −0.002, P = 0.32).

The binary variable is sufficient for prediction, and the main model remains unchanged.
