# Supplementary material for: An interpretable machine learning model for predicting postoperative hypotension in type 2 diabetes mellitus undergoing non‑cardiac surgery
Source: Cardiovasc Diabetol. 2026 Jun 22;25:265. doi: 10.1186/s12933-026-03256-3 (PMC13536729; doi:10.1186/s12933-026-03256-3)
Supplement: Supplementary file 8 [file 12933_2026_3256_MOESM17_ESM.docx]

Supplementary File 14: Validation of PPV/SVV Imputation and Comparison of Invasive Monitoring Subgroups

Background

PPV and SVV were only available for patients undergoing invasive arterial blood pressure monitoring. To assess the impact of missing data and validate the imputation approach, we compared patients with and without invasive monitoring and evaluated model performance using complete‑case analysis vs. multiple imputation.

Methods

Imputation: Multiple imputation by chained equations (MICE) with 20 imputations, using age, ASA class, surgical type, surgical duration, and other haemodynamic variables as predictors.

Complete‑case analysis: Model evaluated only on patients with invasive monitoring (no imputation).

Results

Table. Combined analysis of invasive monitoring subgroups and model performance

|  | Invasive monitoring | No invasive monitoring | P value |
| --- | --- | --- | --- |
| Sample size (retrospective) | 12,834 (42.2%) | 17,578 (57.8%) | – |
| ASA III/IV (%) | 68% | 42% | <0.001 |
| Surgical duration (min, mean ± SD) | 245 ± 98 | 128 ± 65 | <0.001 |
| POH rate (%) | 31% | 19% | <0.001 |
|  | Complete‑case analysis | Multiple imputation | Difference |
| AUC (95% CI) | 0.859 (0.852–0.866) | 0.853 (0.847–0.859) | –0.006 |
| Sensitivity | 0.933 | 0.926 | –0.007 |
| Specificity | 0.521 | 0.516 | –0.005 |

Interpretation

Patients with invasive monitoring had higher ASA scores, longer surgical duration, and higher POH rates, indicating they represent a higher‑risk subgroup. The model performance was nearly identical between complete‑case and imputed analyses (AUC difference –0.006), confirming that multiple imputation did not introduce substantial bias and preserved the full cohort’s representativeness.
