# Supplementary material for: An interpretable machine learning model for predicting postoperative hypotension in type 2 diabetes mellitus undergoing non‑cardiac surgery
Source: Cardiovasc Diabetol. 2026 Jun 22;25:265. doi: 10.1186/s12933-026-03256-3 (PMC13536729; doi:10.1186/s12933-026-03256-3)
Supplement: Supplementary file 9 [file 12933_2026_3256_MOESM18_ESM.docx]

1. Data Preprocessing

All preprocessing steps were defined prior to any analysis and applied consistently across the development and validation datasets.

1.1 Missing Data Handling

Continuous variables with missingness <5%: imputed using the median.

Categorical variables with missingness <5%: imputed using the mode.

Variables with missingness >5% were evaluated for exclusion; if retained, multiple imputation by chained equations (MICE) with 20 imputations was used.

The outcome (POH) had no missing values.

1.2 Outlier Treatment

Outliers were identified using the interquartile range (IQR) rule (values below Q1 − 1.5×IQR or above Q3 + 1.5×IQR).

Extreme outliers were winsorised to the nearest value within the 1.5×IQR range.

1.3 Data Standardisation

All continuous predictors were standardised to Z‑scores (mean = 0, standard deviation = 1) after imputation and outlier treatment, using means and SDs derived from the training set only.

2. Feature Selection

A four‑step feature selection process was pre‑specified to be performed exclusively on the training set using 10‑fold cross‑validation.

Step 1: Univariate Screening

Logistic regression was applied to each candidate predictor.

Variables with p < 0.05 were retained for further analysis.

Step 2: Multicollinearity Assessment

Variance inflation factor (VIF) was calculated for all variables retained in Step 1.

Variables with VIF ≥ 10 were considered highly collinear and removed iteratively until all remaining variables had VIF < 10.

Step 3: Multi‑Algorithm Importance Ranking

Eight algorithms were used to rank feature importance:

Boruta (random forest wrapper)

XGBoost (gain‑based importance)

Random Forest (mean decrease in Gini impurity)

Logistic regression (absolute coefficient)

LASSO (coefficient magnitude)

GBDT (gain‑based importance)

KNN (recursive feature elimination wrapper)

Neural network (connection weights)

A unified importance score was derived by averaging ranks across algorithms.

Step 4: Recursive Feature Elimination (RFE)

Starting with all features that passed Step 2 (n = 57), RFE was performed using random forest with 10‑fold cross‑validation.

At each iteration, the least important feature (based on mean decrease in Gini) was removed.

Performance metrics (AUC, sensitivity, specificity) were recorded at each step.

The final feature set was selected as the smallest number of features at the end of the stable AUC plateau (12–15 features) to balance complexity and generalisability.

3. Model Development and Training

3.1 Data Splitting

Retrospective cohort (N = 34,012 after exclusions) was randomly split into:

Training set: 70% (n = 23,808)

Internal validation set: 30% (n = 10,204)

Prospective cohort (N = 10,528 after exclusions) served as a temporal external validation set, used only after final model locking.

3.2 Models and Hyperparameters

Fourteen models were pre‑specified with fixed hyperparameter grids for tuning:

| Model | Hyperparameters Tuned |
| --- | --- |
| Logistic regression | Penalty (L1/L2), C |
| LASSO | Alpha |
| Random forest | n_estimators, max_depth, min_samples_split |
| XGBoost | n_estimators, max_depth, learning_rate |
| GBDT | n_estimators, max_depth, learning_rate |
| AdaBoost | n_estimators, learning_rate |
| KNN | n_neighbors, weights |
| Naïve Bayes | – |
| Neural network | hidden_layer_size, alpha |
| LDA | – |
| QDA | – |
| SVM | C, gamma |
| Stacking (various combinations) | meta‑learner (logistic regression) |

3.3 Cross‑Validation Strategy

10‑fold cross‑validation was used for hyperparameter tuning and internal performance estimation.

The same fold splits were applied across all models to ensure comparability.

4. Model Performance Evaluation

All performance metrics were pre‑specified and calculated with 95% confidence intervals using bootstrap resampling (1,000 replicates).

| Metric | Definition / Formula | Rationale |
| --- | --- | --- |
| AUC | Area under the receiver operating characteristic curve | Overall discrimination |
| Sensitivity | True positives / (True positives + False negatives) | Ability to detect POH |
| Specificity | True negatives / (True negatives + False positives) | Ability to rule out POH |
| Precision | True positives / (True positives + False positives) | Positive predictive value |
| F1‑score | 2 × (Precision × Sensitivity) / (Precision + Sensitivity) | Harmonic mean of precision and sensitivity |
| Brier score | Mean squared difference between predicted probability and actual outcome | Calibration |
| Calibration curve | Plot of observed vs. predicted probabilities | Visual assessment of calibration |
| Decision curve analysis | Net benefit across threshold probabilities | Clinical utility |

5. Model Interpretability

SHapley Additive exPlanations (SHAP) were pre‑specified as the interpretability framework.

5.1 Global Interpretation

Summary plot: Mean absolute SHAP values to rank feature importance.

Beeswarm plot: Distribution of SHAP values for each feature, coloured by feature value.

5.2 Local Interpretation

Force plots: Visualise how each feature contributes to an individual patient’s risk score.

Waterfall plots: Quantify the contribution of each feature relative to baseline.

6. Subgroup Analyses

Pre‑specified subgroup analyses were planned to assess model robustness:

Subgroup	Rationale

Elective vs. emergency surgery	Different physiology and baseline risk

Patients with vs. without invasive arterial monitoring	PPV/SVV only available in monitored patients

Patients with vs. without heart failure	Different haemodynamic profiles

Performance metrics (AUC, calibration) will be reported separately for each subgroup.

7. Software and Reproducibility

All analyses were conducted using R version 4.5.2 with the following packages:

tidyverse, ggplot2 for data manipulation and visualisation

caret, randomForest, xgboost, glmnet, kernlab, naivebayes for modelling

Boruta, rfe for feature selection

shap for SHAP analysis

pROC, ResourceSelection, rmda for performance evaluation

The full analysis code is publicly available at: https://github.com/mikelu1997/GY

8. Deviations from SAP

No deviations from this pre‑specified plan occurred during the analysis. All analyses were conducted exactly as outlined above.
