# Supplementary material for: An interpretable machine learning model for predicting postoperative hypotension in type 2 diabetes mellitus undergoing non‑cardiac surgery
Source: Cardiovasc Diabetol. 2026 Jun 22;25:265. doi: 10.1186/s12933-026-03256-3 (PMC13536729; doi:10.1186/s12933-026-03256-3)
Supplement: Supplementary file 10 [file 12933_2026_3256_MOESM1_ESM.docx]

| Supplementary Table S11. Variable definitions, units, timing, and sources | | | | |
| --- | --- | --- | --- | --- |
| Variable | Definition | Unit | Time of measurement | Data source |
| Demographics | | | | |
| Age | Patient age at surgery | years | Preoperative | EHR |
| Male sex | Biological sex (male/female) | binary | Preoperative | EHR |
| Body mass index (BMI) | Weight (kg) / Height (m)² | kg/m² | Preoperative | EHR |
| Comorbidities | | | | |
| Hypertension | Documented diagnosis of hypertension | binary | Preoperative | EHR (ICD‑10: I10–I15) |
| Long‑standing diabetes (>10 years) or microvascular complications | Diabetes duration >10 years, or presence of diabetic retinopathy/nephropathy | binary | Preoperative | EHR + ophthalmology/nephrology records |
| Coronary artery disease | Documented CAD | binary | Preoperative | EHR (ICD‑10: I20–I25) |
| Congestive heart failure | Documented CHF | binary | Preoperative | EHR (ICD‑10: I50) |
| Chronic kidney disease | eGFR <60 mL/min/1.73m² for ≥3 months | binary | Preoperative | Laboratory (creatinine, eGFR) |
| Obstructive sleep apnoea (OSA) | Documented OSA diagnosis | binary | Preoperative | EHR (ICD‑10: G47.33) |
| History of stroke | Documented prior ischaemic or haemorrhagic stroke | binary | Preoperative | EHR (ICD‑10: I63, I64) |
| History of cancer | Active or past malignancy (excluding non‑melanoma skin cancer) | binary | Preoperative | EHR (ICD‑10: C00–C97) |
| Liver cirrhosis | Documented cirrhosis (Child‑Pugh A–C) | binary | Preoperative | EHR (ICD‑10: K70.3, K74) |
| Preoperative medications | | | | |
| Beta‑blocker | Use of any beta‑blocker | binary | Preoperative (within 30 days) | Medication list |
| ACEI/ARB | Use of ACE inhibitor or angiotensin receptor blocker | binary | Preoperative (within 30 days) | Medication list |
| Calcium channel blocker | Use of any calcium channel blocker | binary | Preoperative (within 30 days) | Medication list |
| Diuretic | Use of any diuretic (thiazide, loop, potassium‑sparing) | binary | Preoperative (within 30 days) | Medication list |
| Insulin | Use of any insulin preparation | binary | Preoperative (within 30 days) | Medication list |
| Antiplatelet agent | Use of aspirin, clopidogrel, etc. | binary | Preoperative (within 30 days) | Medication list |
| Anticoagulant | Use of warfarin, DOACs, heparin | binary | Preoperative (within 30 days) | Medication list |
| Steroid | Chronic steroid use (≥10 mg/day prednisone equivalent for >30 days) | binary | Preoperative (within 90 days) | Medication list |
| Preoperative vital signs | | | | |
| Systolic blood pressure (SBP) | Preoperative resting SBP | mmHg | Preoperative (day of surgery) | EHR (vital signs) |
| Diastolic blood pressure (DBP) | Preoperative resting DBP | mmHg | Preoperative (day of surgery) | EHR (vital signs) |
| Heart rate (HR) | Preoperative resting HR | bpm | Preoperative (day of surgery) | EHR (vital signs) |
| Preoperative laboratory | | | | |
| Haemoglobin | Hb concentration | g/dL | Preoperative (≤30 days) | Laboratory |
| Haematocrit | Hct percentage | % | Preoperative (≤30 days) | Laboratory |
| Creatinine | Serum creatinine | mg/dL | Preoperative (≤30 days) | Laboratory |
| eGFR | Estimated glomerular filtration rate (CKD‑EPI) | mL/min/1.73m² | Preoperative (≤30 days) | Calculated |
| Albumin | Serum albumin | g/dL | Preoperative (≤30 days) | Laboratory |
| Fasting glucose | Fasting blood glucose | mg/dL | Preoperative (≤30 days) | Laboratory |
| Intraoperative variables | | | | |
| Intraoperative blood loss | Estimated blood loss during surgery | mL | Intraoperative | Anaesthesia record |
| Crystalloid administration | Total volume of crystalloid fluids given | mL | Intraoperative | AIMS (fluid input) |
| Surgical duration | Time from skin incision to closure | minutes | Intraoperative | AIMS |
| Postoperative (PACU) variables | | | | |
| PACU admission vital signs | SBP, DBP, HR, RR, SpO₂ at PACU arrival | various | PACU admission | PACU nursing record |
| 1‑hour change in SBP | SBP at 1 hour minus SBP at PACU admission | mmHg | PACU (within 1 hour) | Vital signs monitor |
| 1‑hour change in HR | HR at 1 hour minus HR at PACU admission | bpm | PACU (within 1 hour) | Vital signs monitor |
| Pain score at PACU admission | Numeric rating scale (0–10) | score | PACU admission | Nursing record |
| Shivering | Presence of shivering during PACU stay | binary | PACU stay | Nursing record |
| Level of respiratory support | Room air, nasal cannula, face mask, NIV, invasive ventilation | categorical | PACU stay | Respiratory chart |
| Vasopressor use (detailed definition) | | | | |
| Vasopressor – agent type | Norepinephrine, epinephrine, dopamine, vasopressin, phenylephrine, metaraminol, ephedrine | categorical | Intraoperative | AIMS (medication administration) |
| Table Legend. This table provides a comprehensive definition for each variable considered in the model development, including demographics, comorbidities, preoperative medications, preoperative vital signs and laboratory tests, intraoperative variables, postoperative (PACU) variables, detailed vasopressor use parameters, and the outcome (postoperative hypotension). Abbreviations: EHR, electronic health record; ICD‑10, International Classification of Diseases, 10th revision; ACEI, angiotensin‑converting enzyme inhibitor; ARB, angiotensin receptor blocker; DOAC, direct oral anticoagulant; eGFR, estimated glomerular filtration rate; AIMS, anaesthesia information management system; PACU, post‑anaesthesia care unit; SBP, systolic blood pressure; DBP, diastolic blood pressure; HR, heart rate; RR, respiratory rate; SpO₂, peripheral oxygen saturation; NIV, non‑invasive ventilation. | | | | |
