# Supplementary material for: An interpretable machine learning model for predicting postoperative hypotension in type 2 diabetes mellitus undergoing non‑cardiac surgery
Source: Cardiovasc Diabetol. 2026 Jun 22;25:265. doi: 10.1186/s12933-026-03256-3 (PMC13536729; doi:10.1186/s12933-026-03256-3)
Supplement: Supplementary file 11 [file 12933_2026_3256_MOESM21_ESM.docx]

Supplementary

Supplementary Table S1. Comprehensive variable definitions, units, measurement timing, and data sources.

Supplementary Table S2. Predictors significantly associated with postoperative hypotension in univariate regression analysis of the training set.

Supplementary Table S3. Results of multicollinearity analysis.

Supplementary Table S4. Feature importance ranking based on multiple machine learning algorithms.

Supplementary Table S5. RFE feature sequence.

Supplementary Table S6. Comprehensive performance of machine learning models on the training set with 95% confidence intervals for AUC.

Supplementary Table S7. Detailed performance metrics of different stacking ensemble model combinations on the training set with 95% confidence intervals for AUC.

Supplementary Table S8. Comprehensive performance of machine learning models on the internal validation set with 95% confidence intervals for AUC.

Supplementary Table S9. Performance of various stacking ensemble combinations on the internal validation set with 95% confidence intervals for AUC.

Supplementary Table S10. Comprehensive performance of machine learning models on the prospective validation set with 95% confidence intervals for AUC.

Supplementary Table S11. Comprehensive performance metrics of the stacking ensemble combinations on the prospective validation set with 95% confidence intervals for AUC.

Supplementary Table S12. Performance of the random forest model on the external validation cohort (No. 991 Hospital, n = 2,156).

Supplementary Table S13. Top 10 feature importance ranks in elective versus emergency surgery subgroups.

Supplementary File 1. TRIPOD + AI reporting checklist.

Supplementary File 2. Sensitivity analysis of heart failure quantification (binary vs. NYHA).

Supplementary File 3. Validation of PPV/SVV imputation.

Supplementary File 4. Statistical Analysis Plan (SAP).

Supplementary File 5. Association between PACU hypotension and ward hypotension.

Supplementary FigureS1. External validation of the random forest model (n = 2,156).

A Calibration curve. The diagonal dashed line indicates perfect calibration. The solid blue line shows actual performance (calibration slope 0.95, intercept − 0.05), indicating good agreement.

B Decision curve analysis. The blue curve shows net benefit of the model across threshold probabilities 0.10–0.45, supporting clinical utility.

Supplementary FigureS2. SHAP Local Interpretability Analysis for Individual Case Predictions.

A-E. SHAP force plots for five representative patient samples, visually demonstrating how each feature's contribution pushes the baseline prediction to the final model output.

F-J. SHAP waterfall plots corresponding to samples A-E, providing a detailed breakdown of the exact contribution value of each feature to the individual prediction.
