# Supplementary material for: An interpretable machine learning model for predicting postoperative hypotension in type 2 diabetes mellitus undergoing non‑cardiac surgery
Source: Cardiovasc Diabetol. 2026 Jun 22;25:265. doi: 10.1186/s12933-026-03256-3 (PMC13536729; doi:10.1186/s12933-026-03256-3)
Supplement: Supplementary file 12 [file 12933_2026_3256_MOESM2_ESM.docx]

Supplementary Table S1. Predictors significantly associated with postoperative hypotension in univariate regression analysis of the training set

| Variable | OR | CI_lower | CI_upper | P_Value |
| --- | --- | --- | --- | --- |
| Age | 1.0501 | 1.0487 | 1.0515 | <0.0001 |
| BMI | 0.8943 | 0.8871 | 0.9016 | <0.0001 |
| ObesityGrade | 2.5644 | 2.5070 | 2.6231 | <0.0001 |
| ASAGrade | 3.5863 | 3.4951 | 3.6799 | <0.0001 |
| CharlsonIndex | 2.9405 | 2.8792 | 3.0031 | <0.0001 |
| Hypertension | 6.8097 | 6.5306 | 7.1007 | <0.0001 |
| Diabetes | 7.1483 | 6.8532 | 7.4560 | <0.0001 |
| HbA1c | 1.1825 | 1.1247 | 1.2431 | <0.0001 |
| CAD | 6.4564 | 6.1938 | 6.7301 | <0.0001 |
| CHF | 7.1805 | 6.8839 | 7.4898 | <0.0001 |
| CKD | 6.3508 | 6.0930 | 6.6194 | <0.0001 |
| COPD | 7.2888 | 6.9870 | 7.6035 | <0.0001 |
| Arrhythmia | 6.7948 | 6.5164 | 7.0851 | <0.0001 |
| Stroke | 8.7101 | 7.9381 | 9.5572 | <0.0001 |
| Cirrhosis | 12.0314 | 10.7707 | 13.4397 | <0.0001 |
| ACEI_ARB | 5.0705 | 4.8693 | 5.2800 | <0.0001 |
| BetaBlocker | 5.0434 | 4.8434 | 5.2517 | <0.0001 |
| Diuretic | 5.7655 | 5.5346 | 6.0061 | <0.0001 |
| Insulin | 5.6941 | 5.4663 | 5.9314 | <0.0001 |
| AntiPlatelet | 5.6993 | 5.4713 | 5.9369 | <0.0001 |
| Anticoagulant | 12.8072 | 11.0099 | 14.8979 | <0.0001 |
| PreopSBP | 0.8934 | 0.8906 | 0.8942 | <0.0001 |
| PreopDBP | 0.8466 | 0.8440 | 0.8491 | <0.0001 |
| PreopMAP | 0.7501 | 0.7450 | 0.7533 | <0.0001 |
| PreopHR | 1.1755 | 1.1721 | 1.1790 | <0.0001 |
| Hb | 0.1992 | 0.1930 | 0.2057 | <0.0001 |
| Hct | 0.6298 | 0.6245 | 0.6352 | <0.0001 |
| Cr | 12.8396 | 11.9693 | 13.7731 | <0.0001 |
| eGFR | 0.9235 | 0.9222 | 0.9249 | <0.0001 |
| BNP | 1.0140 | 1.0137 | 1.0143 | <0.0001 |
| Albumin | 9.6447E-05 | 7.4027E-05 | 1.2565E-04 | <0.0001 |
| Sodium | 0.6310 | 0.6257 | 0.6363 | <0.0001 |
| Potassium | 12.4686 | 11.0783 | 14.0334 | <0.0001 |
| BloodLoss | 1.0029 | 1.0028 | 1.0030 | <0.0001 |
| BPV | 1.9598 | 1.9318 | 1.9883 | <0.0001 |
| IntraopMinHR | 0.9273 | 0.9250 | 0.9295 | <0.0001 |
| IntraopMaxHR | 1.0829 | 1.0811 | 1.0847 | <0.0001 |
| HRV | 1.6852 | 1.6660 | 1.7046 | <0.0001 |
| SVV | 2.2433 | 2.1959 | 2.2917 | <0.0001 |
| PPV | 2.5378 | 2.4622 | 2.6157 | <0.0001 |
| IntraopUrine | 0.9717 | 0.9711 | 0.9722 | <0.0001 |
| MeanBIS | 0.7154 | 0.7110 | 0.7197 | <0.0001 |
| MinBIS | 0.7147 | 0.7101 | 0.7193 | <0.0001 |
| BISUnder40Time | 1.9275 | 1.8748 | 1.9817 | <0.0001 |
| BISVariability | 2.1185 | 2.0865 | 2.1509 | <0.0001 |
| BISHypotensionEvents | 2.9828 | 2.9134 | 3.0538 | <0.0001 |
| PropofolDose | 1.0077 | 1.0075 | 1.0078 | <0.0001 |
| RemifentanilDose | 1.00075 | 1.0007 | 1.0008 | <0.0001 |
| MinTemp | 1.1768E-04 | 7.8733E-05 | 1.7595E-04 | <0.0001 |
| HypothermiaTime | 1.2752 | 1.2689 | 1.2816 | <0.0001 |
| MinETCO2 | 0.5877 | 0.5820 | 0.5934 | <0.0001 |
| MinSpO2 | 0.1558 | 0.1454 | 0.1671 | <0.0001 |
| MinBE | 0.1003 | 0.0946 | 0.1064 | <0.0001 |
| PostopSBP | 0.9158 | 0.9133 | 0.9183 | <0.0001 |
| PostopDBP | 0.8608 | 0.8585 | 0.8632 | <0.0001 |
| PostopMAP | 0.7803 | 0.7757 | 0.7829 | <0.0001 |
| PostopHR | 1.0526 | 1.0509 | 1.0543 | <0.0001 |
| SBPChange1h | 0.6566 | 0.6499 | 0.6613 | <0.0001 |
| HRChange1h | 1.5651 | 1.5522 | 1.5781 | <0.0001 |
| PostopVasopressor | 5.6253 | 5.3321 | 5.9345 | <0.0001 |
| CCB | 3.0933 | 2.9198 | 3.2772 | <0.0001 |
| Crystalloid | 1.0036 | 1.0034 | 1.0038 | <0.0001 |
| OSA | 2.5663 | 2.4357 | 2.7011 | <0.0001 |
| Steroid | 4.4681 | 4.0853 | 4.8868 | <0.0001 |
| Cancer | 2.2493 | 2.1424 | 2.3615 | <0.0001 |
| AnesthesiaDuration | 1.0034 | 1.0031 | 1.0036 | <0.0001 |
| SurgeryDuration | 1.0024 | 1.0022 | 1.0027 | <0.0001 |
| HypotensionAUC | 1.6550 | 1.5749 | 1.7391 | <0.0001 |
| IntraopHypotensionTime | 1.8475 | 1.6941 | 2.0149 | <0.0001 |
| MinPH | 6.4977E-94 | 8.3784E-109 | 5.0391E-79 | <0.0001 |
| VasopressorTime | 1.5784 | 1.4589 | 1.7076 | <0.0001 |
| PeakLactate | 12.1773 | 1.3815 | 107.3385 | <0.0001 |
| IntraopMinMAP | 0.2893 | 0.2173 | 0.3853 | <0.0001 |
| PostopRR | 1.0060 | 1.0003 | 1.0117 | 0.0395 |
