# Supplementary material for: An interpretable machine learning model for predicting postoperative hypotension in type 2 diabetes mellitus undergoing non‑cardiac surgery
Source: Cardiovasc Diabetol. 2026 Jun 22;25:265. doi: 10.1186/s12933-026-03256-3 (PMC13536729; doi:10.1186/s12933-026-03256-3)
Supplement: Supplementary file 13 [file 12933_2026_3256_MOESM3_ESM.docx]

TableS2.Results of Multicollinearity Analysis

| Variable | VIF |
| --- | --- |
| SVV | 9.364 |
| Arrhythmia | 7.925 |
| MeanBIS | 7.693 |
| Steroid | 7.376 |
| HypotensionAUC | 7.367 |
| ASAGrade | 6.956 |
| VasopressorTime | 6.602 |
| PeakLactate | 6.290 |
| CharlsonIndex | 6.089 |
| PPV | 5.504 |
| BPV | 4.923 |
| BISVariability | 4.831 |
| Age | 4.758 |
| MinPH | 4.606 |
| IntraopMinHR | 4.537 |
| Hypertension | 4.531 |
| HypothermiaTime | 4.454 |
| MinSpO2 | 4.396 |
| Albumin | 4.123 |
| AntiPlatelet | 4.072 |
| MinETCO2 | 3.984 |
| BloodLoss | 3.918 |
| COPD | 3.639 |
| MinBE | 3.619 |
| Cancer | 3.411 |
| IntraopMaxHR | 3.209 |
| Insulin | 3.017 |
| CAD | 2.586 |
| MinBIS | 2.422 |
| HRChange1h | 2.299 |
| ObesityGrade | 2.215 |
| BNP | 1.956 |
| eGFR | 1.835 |
| OSA | 1.759 |
| BMI | 1.758 |
| ACEI_ARB | 1.750 |
| Crystalloid | 1.726 |
| Potassium | 1.669 |
| Cr | 1.646 |
| CHF | 1.637 |
| PropofolDose | 1.592 |
| Cirrhosis | 1.586 |
| Diabetes | 1.528 |
| Stroke | 1.450 |
| HbA1c | 1.42 |
| Sodium | 1.361 |
| PostopVasopressor | 1.298 |
| RemifentanilDose | 1.294 |
| PostopRR | 1.279 |
| Diuretic | 1.261 |
| CCB | 1.240 |
| CKD | 1.193 |
| BetaBlocker | 1.156 |
| Anticoagulant | 1.060 |
| BISHypotensionEvents | 1.057 |
| HRV | 1.007 |
| BISUnder40Time | 1.004 |
