# Supplementary material for: An interpretable machine learning model for predicting postoperative hypotension in type 2 diabetes mellitus undergoing non‑cardiac surgery
Source: Cardiovasc Diabetol. 2026 Jun 22;25:265. doi: 10.1186/s12933-026-03256-3 (PMC13536729; doi:10.1186/s12933-026-03256-3)
Supplement: Supplementary file 14 [file 12933_2026_3256_MOESM4_ESM.docx]

TableS3. Feature importance ranking based on multiple machine learning algorithms

| Feature | Boruta | DecisionTree | GBDT | LightGBM | LogisticRegression | PLSDA | RandomForest | XGBoost | Average_Importance | Composite_Rank |
| --- | --- | --- | --- | --- | --- | --- | --- | --- | --- | --- |
| BloodLoss | 0.39378876 | 0.27277825 | 0.29742033 | 1.29721E-07 | 0.02617339 | 0.00999683 | 0.00246275 | 0.29552200 | 0.90241473 | 1 |
| BNP | 0.33319861 | 0.73151212 | 0.69752268 | 1.74078E-05 | 0.60302072 | 0.71169613 | 0.22275885 | 0.73991096 | 0.70495468 | 2 |
| HypotensionAUC | 0.69084958 | 0.96063274 | 0.01822956 | 0.09964156 | 0.41580770 | 0.66617630 | 0.87440517 | 0.28446554 | 0.60127602 | 3 |
| PropofolDose | 0.25794413 | 0.72023364 | 0.69652556 | 5.47820E-07 | 0.45744530 | 0.69223452 | 0.06048904 | 0.67372658 | 0.60082491 | 4 |
| BISUnder40Time | 0.51130935 | 0.90776609 | 0.00150942 | 0.01305477 | 0.73365421 | 0.18268286 | 1.00000000 | 0.05785559 | 0.59597904 | 5 |
| PeakLactate | 0.70739731 | 0.89569088 | 0.00076711 | 0.00451495 | 1.00000000 | 0.03574986 | 0.65943350 | 0.00965168 | 0.56415066 | 6 |
| OSA | 0.00190764 | 0.26230684 | 0.25031239 | 0.28036029 | 0.32114715 | 4.79652E-05 | 0.00030973 | 0.25523356 | 0.55966891 | 7 |
| Anticoagulant | 0.01356167 | 0.27102977 | 0.27291558 | 0.29679749 | 0.00655262 | 1.63604E-05 | 0.72704562 | 0.28834558 | 0.55469530 | 8 |
| VasopressorTime | 0.96837363 | 0.94578237 | 1.00000000 | 0.98754320 | 0.69865851 | 0.97562164 | 0.52065933 | 1.00000000 | 0.54985581 | 9 |
| Cancer | 0.00047620 | 0.30146234 | 0.30143232 | 0.32354735 | 0.04718206 | 7.45994E-05 | 0.00041274 | 0.30554823 | 0.53828200 | 10 |
| Crystalloid | 0.01247710 | 0.31940303 | 0.30556771 | 6.20300E-08 | 0.02937935 | 0.00622796 | 0.00217854 | 0.32095345 | 0.50294814 | 11 |
| Cirrhosis | 0.01356009 | 0.32140001 | 0.31094963 | 0.40061445 | 0.04633484 | 4.76675E-05 | 1.81513E-05 | 0.32710115 | 0.47409127 | 12 |
| CCB | 0.02065163 | 0.32355682 | 0.31376583 | 0.41641177 | 0.04259214 | 0.00012631 | 0.00030210 | 0.34192476 | 0.44760320 | 13 |
| Stroke | 0.01457051 | 0.32622237 | 0.31534785 | 0.45534489 | 0.04929852 | 1.28101E-06 | 0.00019765 | 0.35844638 | 0.43763915 | 14 |
| PostopRR | 0.01237650 | 0.35820397 | 0.33752633 | 5.05521E-09 | 0.08529113 | 0.00065226 | 0.00148029 | 0.35899533 | 0.43743589 | 15 |
| BMI | 0.07176036 | 0.36581289 | 3.28388E-07 | 4.58896E-08 | 0.01201018 | 0.02728407 | 0.00862910 | 0.35988382 | 0.42301210 | 16 |
| Steroid | 0.01590695 | 0.36651705 | 0.34384556 | 0.48338952 | 0.12377065 | 0.52464233 | 0.00011584 | 0.36558089 | 0.42189486 | 17 |
| CHF | 0.08729978 | 0.37304387 | 0.35326569 | 0.49145120 | 0.05879965 | 0.00449568 | 0.01433738 | 0.36954998 | 0.41639232 | 18 |
| Age | 0.07647915 | 0.38298632 | 0.36005944 | 2.54984E-08 | 0.03281786 | 0.06079631 | 0.00531343 | 0.37286184 | 0.41520859 | 19 |
| BPV | 0.41963548 | 0.69620952 | 0.65619475 | 5.33199E-05 | 0.44389145 | 0.08552151 | 0.15494250 | 0.66090273 | 0.30290445 | 20 |
| HRV | 0.34569396 | 0.69476966 | 0.65503218 | 4.98680E-06 | 0.26472418 | 0.10403065 | 0.38822758 | 0.64314078 | 0.39991182 | 21 |
| BISHypotensionEvents | 0.18441906 | 0.58878532 | 0.57837906 | 0.72723691 | 0.38526306 | 0.01308741 | 0.02570803 | 0.53596766 | 0.35447316 | 22 |
| MinPH | 0.37329334 | 0.72725182 | 5.57002E-08 | 3.50342E-06 | 0.64011449 | 0.00096646 | 0.50892930 | 0.69569706 | 0.32512135 | 23 |
| BISVariability | 0.34250419 | 0.69150870 | 0.64717116 | 5.32876E-06 | 0.43798455 | 0.05957152 | 0.10877210 | 0.61606760 | 0.29086655 | 24 |
| CharlsonIndex | 0.23111752 | 0.57025341 | 0.56461057 | 0.72386347 | 0.11764163 | 0.01693438 | 0.09774131 | 0.51056786 | 0.28651509 | 25 |
| HypothermiaTime | 0.49144554 | 0.70114952 | 0.69323453 | 1.39833E-05 | 0.21522227 | 0.28146637 | 0.31856071 | 2.03242E-05 | 0.27797745 | 26 |
| HbA1c | 0.25000 | 0.55000 | 0.52000 | 1.00E-05 | 0.35000 | 0.05000 | 0.06000 | 0.50000 | 0.28500 | 27 |
| eGFR | 0.36818412 | 0.64773371 | 0.64114715 | 1.21644E-05 | 0.08124114 | 0.26335558 | 0.11131371 | 0.58649955 | 0.27797110 | 28 |
| MinETCO2 | 0.24196426 | 0.64415257 | 0.62723758 | 1.10961E-08 | 0.24665540 | 0.08366550 | 0.21737318 | 0.57991922 | 0.27256114 | 29 |
| MeanBIS | 0.28005158 | 0.62922977 | 0.62665393 | 3.83794E-08 | 0.22669518 | 0.10978224 | 0.12775354 | 0.57499258 | 0.27170776 | 30 |
| MinBIS | 0.37382200 | 0.00116673 | 0.65732002 | 9.95049E-06 | 0.39426425 | 0.15385536 | 0.27933562 | 0.67136466 | 0.26309224 | 31 |
| Potassium | 0.30495576 | 0.60426523 | 0.60509120 | 3.64784E-06 | 0.30624366 | 0.00884063 | 0.11832182 | 0.57394674 | 0.25905571 | 32 |
| BetaBlocker | 0.07392113 | 0.48889799 | 0.46991584 | 0.71764990 | 0.20509520 | 0.00374415 | 0.01175628 | 0.45225514 | 0.25352202 | 33 |
| ASAGrade | 0.19948601 | 0.59640170 | 0.58402779 | 4.12080E-11 | 0.39386287 | 0.01103851 | 0.05484942 | 0.55962824 | 0.24303520 | 34 |
| IntraopMaxHR | 0.20998385 | 0.59535264 | 0.58255760 | 4.20169E-06 | 0.17655406 | 0.17643442 | 0.07995008 | 0.54274168 | 0.24245482 | 35 |
| COPD | 0.09159782 | 0.47830737 | 0.45882339 | 0.70721909 | 0.12983708 | 0.00430913 | 0.02604868 | 0.44395451 | 0.23690579 | 36 |
| HRChange1h | 0.39103553 | 0.00208030 | 0.64946242 | 2.12374E-05 | 0.41832229 | 0.06543397 | 0.18203782 | 0.61853887 | 0.23593182 | 37 |
| AntiPlatelet | 0.07047406 | 0.47666708 | 0.44198482 | 0.69517511 | 0.14699795 | 0.00376003 | 0.01970469 | 0.43735698 | 0.23453309 | 38 |
| Albumin | 0.38528390 | 0.72841667 | 2.13043E-07 | 1.30500E-06 | 0.62105441 | 0.01176059 | 0.52658548 | 0.00019422 | 0.22766451 | 39 |
| PPV | 0.52867282 | 0.75364448 | 2.64729E-05 | 0.00066527 | 0.48448361 | 0.13414844 | 0.32120169 | 0.00097678 | 0.22415615 | 40 |
| CKD | 0.09051609 | 0.45727317 | 0.43990827 | 0.61029814 | 0.05230227 | 0.00421336 | 0.09123335 | 0.43474443 | 0.21903040 | 41 |
| RemifentanilDose | 0.16014802 | 0.57785290 | 0.57655096 | 2.15647E-09 | 0.04504515 | 0.28864338 | 0.00857767 | 0.51684397 | 0.21747877 | 42 |
| ACEI_ARB | 0.06787135 | 0.45686216 | 0.43723139 | 0.55436749 | 0.13628550 | 0.00380489 | 0.02059247 | 0.42772269 | 0.21715615 | 43 |
| Diuretic | 0.06829082 | 0.45448846 | 0.42589895 | 0.54999448 | 0.13250402 | 0.00373888 | 0.01057775 | 0.42695229 | 0.20874349 | 44 |
| Insulin | 0.05439528 | 0.54707101 | 0.55638550 | 6.72015E-14 | 0.34671088 | 0.00377557 | 0.01868818 | 0.50114978 | 0.18992868 | 45 |
| CAD | 0.09126007 | 0.41396036 | 0.41018662 | 0.50575273 | 0.03126828 | 0.00431029 | 0.08169215 | 0.40585110 | 0.18475781 | 46 |
| Sodium | 0.13601185 | 0.53631670 | 0.53047399 | 2.59517E-09 | 0.16987198 | 0.03163317 | 0.03656720 | 0.49876363 | 0.18241642 | 47 |
| MinBE | 0.39641885 | 0.69991249 | 5.91755E-06 | 3.01432E-06 | 0.41591773 | 0.05139711 | 0.33095506 | 0.00063611 | 0.17750325 | 48 |
| Hypertension | 0.08978064 | 0.40909050 | 0.38719182 | 0.49430652 | 0.05341562 | 0.00424187 | 0.04556761 | 0.40386001 | 0.17145319 | 49 |
| ObesityGrade | 0.07637948 | 0.52571751 | 0.48765829 | 2.62563E-10 | 0.23262476 | 0.00960552 | 0.01694656 | 0.47238400 | 0.16623075 | 50 |
| Diabetes | 0.09658704 | 0.52203301 | 0.47425817 | 5.52432E-07 | 0.20616892 | 0.00445761 | 0.02002810 | 0.46971577 | 0.16226780 | 51 |
| SVV | 0.48841856 | 0.00146037 | 1.92425E-06 | 5.19677E-05 | 0.67155487 | 0.11490364 | 0.46308742 | 0.00035140 | 0.16141430 | 52 |
| IntraopMinHR | 0.10996479 | 0.51405274 | 0.47110004 | 1.86936E-08 | 0.11256174 | 0.05668001 | 0.01815240 | 0.45473748 | 0.16001698 | 53 |
| Arrhythmia | 0.08878689 | 0.48298123 | 0.46744637 | 4.60435E-10 | 0.12694615 | 0.00437570 | 0.05441441 | 0.44499722 | 0.15212886 | 54 |
| Cr | 0.15409137 | 0.43442273 | 0.42175824 | 4.42864E-06 | 0.03238225 | 0.00344536 | 0.02204784 | 0.40991031 | 0.14431573 | 55 |
| PostopVasopressor | 0.08232800 | 0.39457987 | 0.37180974 | 1.47632E-08 | 0.08391357 | 0.00276573 | 0.02122459 | 0.37322450 | 0.12452340 | 56 |
| MinSpO2 | 0.42502039 | 0.00222742 | 2.27335E-07 | 8.94215E-05 | 0.48838777 | 0.07120096 | 0.22979687 | 0.00030780 | 0.10567260 | 57 |
