# Supplementary material for: An interpretable machine learning model for predicting postoperative hypotension in type 2 diabetes mellitus undergoing non‑cardiac surgery
Source: Cardiovasc Diabetol. 2026 Jun 22;25:265. doi: 10.1186/s12933-026-03256-3 (PMC13536729; doi:10.1186/s12933-026-03256-3)
Supplement: Supplementary file 15 [file 12933_2026_3256_MOESM5_ESM.docx]

Table S4. RFE Feature Sequence

| NumFeatures | Features (ordered from least to most important at each step) |
| --- | --- |
| 57 | VasopressorTime, HypotensionAUC, BISUnder40Time, PeakLactate, PPV, BNP, SVV, Albumin, MinPH, PropofolDose, HypothermiaTime, MinSpO2, MinBIS, MinBE, BPV, HRV, HRChange1h, BISVariability, eGFR, MinETCO2, MeanBIS, Potassium, ASAGrade, IntraopMaxHR, BISHypotensionEvents, RemifentanilDose, CharlsonIndex, Insulin, Sodium, ObesityGrade, Diabetes, IntraopMinHR, BetaBlocker, HbA1c, Arrhythmia, COPD, AntiPlatelet, CKD, ACEI_ARB, Diuretic, Cr, CAD, Hypertension, PostopVasopressor, Age, CHF, Steroid, BMI, PostopRR, Stroke, CCB, Cirrhosis, Crystalloid, Cancer, BloodLoss, Anticoagulant, OSA |
| 56 | HypotensionAUC, BISUnder40Time, PeakLactate, PPV, BNP, SVV, Albumin, MinPH, PropofolDose, HypothermiaTime, MinSpO2, MinBIS, MinBE, BPV, HRV, HRChange1h, BISVariability, eGFR, MinETCO2, MeanBIS, Potassium, ASAGrade, IntraopMaxHR, BISHypotensionEvents, RemifentanilDose, CharlsonIndex, Insulin, Sodium, ObesityGrade, Diabetes, IntraopMinHR, BetaBlocker, HbA1c, Arrhythmia, COPD, AntiPlatelet, CKD, ACEI_ARB, Diuretic, Cr, CAD, Hypertension, PostopVasopressor, Age, CHF, Steroid, BMI, PostopRR, Stroke, CCB, Cirrhosis, Crystalloid, Cancer, BloodLoss, Anticoagulant, OSA |
| 55 | BISUnder40Time, PeakLactate, PPV, BNP, SVV, Albumin, MinPH, PropofolDose, HypothermiaTime, MinSpO2, MinBIS, MinBE, BPV, HRV, HRChange1h, BISVariability, eGFR, MinETCO2, MeanBIS, Potassium, ASAGrade, IntraopMaxHR, BISHypotensionEvents, RemifentanilDose, CharlsonIndex, Insulin, Sodium, ObesityGrade, Diabetes, IntraopMinHR, BetaBlocker, HbA1c, Arrhythmia, COPD, AntiPlatelet, CKD, ACEI_ARB, Diuretic, Cr, CAD, Hypertension, PostopVasopressor, Age, CHF, Steroid, BMI, PostopRR, Stroke, CCB, Cirrhosis, Crystalloid, Cancer, BloodLoss, Anticoagulant, OSA |
| 54 | PeakLactate, PPV, BNP, SVV, Albumin, MinPH, PropofolDose, HypothermiaTime, MinSpO2, MinBIS, MinBE, BPV, HRV, HRChange1h, BISVariability, eGFR, MinETCO2, MeanBIS, Potassium, ASAGrade, IntraopMaxHR, BISHypotensionEvents, RemifentanilDose, CharlsonIndex, Insulin, Sodium, ObesityGrade, Diabetes, IntraopMinHR, BetaBlocker, HbA1c, Arrhythmia, COPD, AntiPlatelet, CKD, ACEI_ARB, Diuretic, Cr, CAD, Hypertension, PostopVasopressor, Age, CHF, Steroid, BMI, PostopRR, Stroke, CCB, Cirrhosis, Crystalloid, Cancer, BloodLoss, Anticoagulant, OSA |
| 53 | PPV, BNP, SVV, Albumin, MinPH, PropofolDose, HypothermiaTime, MinSpO2, MinBIS, MinBE, BPV, HRV, HRChange1h, BISVariability, eGFR, MinETCO2, MeanBIS, Potassium, ASAGrade, IntraopMaxHR, BISHypotensionEvents, RemifentanilDose, CharlsonIndex, Insulin, Sodium, ObesityGrade, Diabetes, IntraopMinHR, BetaBlocker, HbA1c, Arrhythmia, COPD, AntiPlatelet, CKD, ACEI_ARB, Diuretic, Cr, CAD, Hypertension, PostopVasopressor, Age, CHF, Steroid, BMI, PostopRR, Stroke, CCB, Cirrhosis, Crystalloid, Cancer, BloodLoss, Anticoagulant, OSA |
| 52 | BNP, SVV, Albumin, MinPH, PropofolDose, HypothermiaTime, MinSpO2, MinBIS, MinBE, BPV, HRV, HRChange1h, BISVariability, eGFR, MinETCO2, MeanBIS, Potassium, ASAGrade, IntraopMaxHR, BISHypotensionEvents, RemifentanilDose, CharlsonIndex, Insulin, Sodium, ObesityGrade, Diabetes, IntraopMinHR, BetaBlocker, HbA1c, Arrhythmia, COPD, AntiPlatelet, CKD, ACEI_ARB, Diuretic, Cr, CAD, Hypertension, PostopVasopressor, Age, CHF, Steroid, BMI, PostopRR, Stroke, CCB, Cirrhosis, Crystalloid, Cancer, BloodLoss, Anticoagulant, OSA |
| 51 | SVV, Albumin, MinPH, PropofolDose, HypothermiaTime, MinSpO2, MinBIS, MinBE, BPV, HRV, HRChange1h, BISVariability, eGFR, MinETCO2, MeanBIS, Potassium, ASAGrade, IntraopMaxHR, BISHypotensionEvents, RemifentanilDose, CharlsonIndex, Insulin, Sodium, ObesityGrade, Diabetes, IntraopMinHR, BetaBlocker, HbA1c, Arrhythmia, COPD, AntiPlatelet, CKD, ACEI_ARB, Diuretic, Cr, CAD, Hypertension, PostopVasopressor, Age, CHF, Steroid, BMI, PostopRR, Stroke, CCB, Cirrhosis, Crystalloid, Cancer, BloodLoss, Anticoagulant, OSA |
| 50 | Albumin, MinPH, PropofolDose, HypothermiaTime, MinSpO2, MinBIS, MinBE, BPV, HRV, HRChange1h, BISVariability, eGFR, MinETCO2, MeanBIS, Potassium, ASAGrade, IntraopMaxHR, BISHypotensionEvents, RemifentanilDose, CharlsonIndex, Insulin, Sodium, ObesityGrade, Diabetes, IntraopMinHR, BetaBlocker, HbA1c, Arrhythmia, COPD, AntiPlatelet, CKD, ACEI_ARB, Diuretic, Cr, CAD, Hypertension, PostopVasopressor, Age, CHF, Steroid, BMI, PostopRR, Stroke, CCB, Cirrhosis, Crystalloid, Cancer, BloodLoss, Anticoagulant, OSA |
| 49 | MinPH, PropofolDose, HypothermiaTime, MinSpO2, MinBIS, MinBE, BPV, HRV, HRChange1h, BISVariability, eGFR, MinETCO2, MeanBIS, Potassium, ASAGrade, IntraopMaxHR, BISHypotensionEvents, RemifentanilDose, CharlsonIndex, Insulin, Sodium, ObesityGrade, Diabetes, IntraopMinHR, BetaBlocker, HbA1c, Arrhythmia, COPD, AntiPlatelet, CKD, ACEI_ARB, Diuretic, Cr, CAD, Hypertension, PostopVasopressor, Age, CHF, Steroid, BMI, PostopRR, Stroke, CCB, Cirrhosis, Crystalloid, Cancer, BloodLoss, Anticoagulant, OSA |
| 48 | PropofolDose, HypothermiaTime, MinSpO2, MinBIS, MinBE, BPV, HRV, HRChange1h, BISVariability, eGFR, MinETCO2, MeanBIS, Potassium, ASAGrade, IntraopMaxHR, BISHypotensionEvents, RemifentanilDose, CharlsonIndex, Insulin, Sodium, ObesityGrade, Diabetes, IntraopMinHR, BetaBlocker, HbA1c, Arrhythmia, COPD, AntiPlatelet, CKD, ACEI_ARB, Diuretic, Cr, CAD, Hypertension, PostopVasopressor, Age, CHF, Steroid, BMI, PostopRR, Stroke, CCB, Cirrhosis, Crystalloid, Cancer, BloodLoss, Anticoagulant, OSA |
| 47 | HypothermiaTime, MinSpO2, MinBIS, MinBE, BPV, HRV, HRChange1h, BISVariability, eGFR, MinETCO2, MeanBIS, Potassium, ASAGrade, IntraopMaxHR, BISHypotensionEvents, RemifentanilDose, CharlsonIndex, Insulin, Sodium, ObesityGrade, Diabetes, IntraopMinHR, BetaBlocker, HbA1c, Arrhythmia, COPD, AntiPlatelet, CKD, ACEI_ARB, Diuretic, Cr, CAD, Hypertension, PostopVasopressor, Age, CHF, Steroid, BMI, PostopRR, Stroke, CCB, Cirrhosis, Crystalloid, Cancer, BloodLoss, Anticoagulant, OSA |
| 46 | MinSpO2, MinBIS, MinBE, BPV, HRV, HRChange1h, BISVariability, eGFR, MinETCO2, MeanBIS, Potassium, ASAGrade, IntraopMaxHR, BISHypotensionEvents, RemifentanilDose, CharlsonIndex, Insulin, Sodium, ObesityGrade, Diabetes, IntraopMinHR, BetaBlocker, HbA1c, Arrhythmia, COPD, AntiPlatelet, CKD, ACEI_ARB, Diuretic, Cr, CAD, Hypertension, PostopVasopressor, Age, CHF, Steroid, BMI, PostopRR, Stroke, CCB, Cirrhosis, Crystalloid, Cancer, BloodLoss, Anticoagulant, OSA |
| 45 | MinBIS, MinBE, BPV, HRV, HRChange1h, BISVariability, eGFR, MinETCO2, MeanBIS, Potassium, ASAGrade, IntraopMaxHR, BISHypotensionEvents, RemifentanilDose, CharlsonIndex, Insulin, Sodium, ObesityGrade, Diabetes, IntraopMinHR, BetaBlocker, HbA1c, Arrhythmia, COPD, AntiPlatelet, CKD, ACEI_ARB, Diuretic, Cr, CAD, Hypertension, PostopVasopressor, Age, CHF, Steroid, BMI, PostopRR, Stroke, CCB, Cirrhosis, Crystalloid, Cancer, BloodLoss, Anticoagulant, OSA |
| 44 | MinBE, BPV, HRV, HRChange1h, BISVariability, eGFR, MinETCO2, MeanBIS, Potassium, ASAGrade, IntraopMaxHR, BISHypotensionEvents, RemifentanilDose, CharlsonIndex, Insulin, Sodium, ObesityGrade, Diabetes, IntraopMinHR, BetaBlocker, HbA1c, Arrhythmia, COPD, AntiPlatelet, CKD, ACEI_ARB, Diuretic, Cr, CAD, Hypertension, PostopVasopressor, Age, CHF, Steroid, BMI, PostopRR, Stroke, CCB, Cirrhosis, Crystalloid, Cancer, BloodLoss, Anticoagulant, OSA |
| 43 | BPV, HRV, HRChange1h, BISVariability, eGFR, MinETCO2, MeanBIS, Potassium, ASAGrade, IntraopMaxHR, BISHypotensionEvents, RemifentanilDose, CharlsonIndex, Insulin, Sodium, ObesityGrade, Diabetes, IntraopMinHR, BetaBlocker, HbA1c, Arrhythmia, COPD, AntiPlatelet, CKD, ACEI_ARB, Diuretic, Cr, CAD, Hypertension, PostopVasopressor, Age, CHF, Steroid, BMI, PostopRR, Stroke, CCB, Cirrhosis, Crystalloid, Cancer, BloodLoss, Anticoagulant, OSA |
| 42 | HRV, HRChange1h, BISVariability, eGFR, MinETCO2, MeanBIS, Potassium, ASAGrade, IntraopMaxHR, BISHypotensionEvents, RemifentanilDose, CharlsonIndex, Insulin, Sodium, ObesityGrade, Diabetes, IntraopMinHR, BetaBlocker, HbA1c, Arrhythmia, COPD, AntiPlatelet, CKD, ACEI_ARB, Diuretic, Cr, CAD, Hypertension, PostopVasopressor, Age, CHF, Steroid, BMI, PostopRR, Stroke, CCB, Cirrhosis, Crystalloid, Cancer, BloodLoss, Anticoagulant, OSA |
| 41 | HRChange1h, BISVariability, eGFR, MinETCO2, MeanBIS, Potassium, ASAGrade, IntraopMaxHR, BISHypotensionEvents, RemifentanilDose, CharlsonIndex, Insulin, Sodium, ObesityGrade, Diabetes, IntraopMinHR, BetaBlocker, HbA1c, Arrhythmia, COPD, AntiPlatelet, CKD, ACEI_ARB, Diuretic, Cr, CAD, Hypertension, PostopVasopressor, Age, CHF, Steroid, BMI, PostopRR, Stroke, CCB, Cirrhosis, Crystalloid, Cancer, BloodLoss, Anticoagulant, OSA |
| 40 | BISVariability, eGFR, MinETCO2, MeanBIS, Potassium, ASAGrade, IntraopMaxHR, BISHypotensionEvents, RemifentanilDose, CharlsonIndex, Insulin, Sodium, ObesityGrade, Diabetes, IntraopMinHR, BetaBlocker, HbA1c, Arrhythmia, COPD, AntiPlatelet, CKD, ACEI_ARB, Diuretic, Cr, CAD, Hypertension, PostopVasopressor, Age, CHF, Steroid, BMI, PostopRR, Stroke, CCB, Cirrhosis, Crystalloid, Cancer, BloodLoss, Anticoagulant, OSA |
| 39 | eGFR, MinETCO2, MeanBIS, Potassium, ASAGrade, IntraopMaxHR, BISHypotensionEvents, RemifentanilDose, CharlsonIndex, Insulin, Sodium, ObesityGrade, Diabetes, IntraopMinHR, BetaBlocker, HbA1c, Arrhythmia, COPD, AntiPlatelet, CKD, ACEI_ARB, Diuretic, Cr, CAD, Hypertension, PostopVasopressor, Age, CHF, Steroid, BMI, PostopRR, Stroke, CCB, Cirrhosis, Crystalloid, Cancer, BloodLoss, Anticoagulant, OSA |
| 38 | MinETCO2, MeanBIS, Potassium, ASAGrade, IntraopMaxHR, BISHypotensionEvents, RemifentanilDose, CharlsonIndex, Insulin, Sodium, ObesityGrade, Diabetes, IntraopMinHR, BetaBlocker, HbA1c, Arrhythmia, COPD, AntiPlatelet, CKD, ACEI_ARB, Diuretic, Cr, CAD, Hypertension, PostopVasopressor, Age, CHF, Steroid, BMI, PostopRR, Stroke, CCB, Cirrhosis, Crystalloid, Cancer, BloodLoss, Anticoagulant, OSA |
| 37 | MeanBIS, Potassium, ASAGrade, IntraopMaxHR, BISHypotensionEvents, RemifentanilDose, CharlsonIndex, Insulin, Sodium, ObesityGrade, Diabetes, IntraopMinHR, BetaBlocker, HbA1c, Arrhythmia, COPD, AntiPlatelet, CKD, ACEI_ARB, Diuretic, Cr, CAD, Hypertension, PostopVasopressor, Age, CHF, Steroid, BMI, PostopRR, Stroke, CCB, Cirrhosis, Crystalloid, Cancer, BloodLoss, Anticoagulant, OSA |
| 36 | Potassium, ASAGrade, IntraopMaxHR, BISHypotensionEvents, RemifentanilDose, CharlsonIndex, Insulin, Sodium, ObesityGrade, Diabetes, IntraopMinHR, BetaBlocker, HbA1c, Arrhythmia, COPD, AntiPlatelet, CKD, ACEI_ARB, Diuretic, Cr, CAD, Hypertension, PostopVasopressor, Age, CHF, Steroid, BMI, PostopRR, Stroke, CCB, Cirrhosis, Crystalloid, Cancer, BloodLoss, Anticoagulant, OSA |
| 35 | ASAGrade, IntraopMaxHR, BISHypotensionEvents, RemifentanilDose, CharlsonIndex, Insulin, Sodium, ObesityGrade, Diabetes, IntraopMinHR, BetaBlocker, HbA1c, Arrhythmia, COPD, AntiPlatelet, CKD, ACEI_ARB, Diuretic, Cr, CAD, Hypertension, PostopVasopressor, Age, CHF, Steroid, BMI, PostopRR, Stroke, CCB, Cirrhosis, Crystalloid, Cancer, BloodLoss, Anticoagulant, OSA |
| 34 | IntraopMaxHR, BISHypotensionEvents, RemifentanilDose, CharlsonIndex, Insulin, Sodium, ObesityGrade, Diabetes, IntraopMinHR, BetaBlocker, HbA1c, Arrhythmia, COPD, AntiPlatelet, CKD, ACEI_ARB, Diuretic, Cr, CAD, Hypertension, PostopVasopressor, Age, CHF, Steroid, BMI, PostopRR, Stroke, CCB, Cirrhosis, Crystalloid, Cancer, BloodLoss, Anticoagulant, OSA |
| 33 | BISHypotensionEvents, RemifentanilDose, CharlsonIndex, Insulin, Sodium, ObesityGrade, Diabetes, IntraopMinHR, BetaBlocker, HbA1c, Arrhythmia, COPD, AntiPlatelet, CKD, ACEI_ARB, Diuretic, Cr, CAD, Hypertension, PostopVasopressor, Age, CHF, Steroid, BMI, PostopRR, Stroke, CCB, Cirrhosis, Crystalloid, Cancer, BloodLoss, Anticoagulant, OSA |
| 32 | RemifentanilDose, CharlsonIndex, Insulin, Sodium, ObesityGrade, Diabetes, IntraopMinHR, BetaBlocker, HbA1c, Arrhythmia, COPD, AntiPlatelet, CKD, ACEI_ARB, Diuretic, Cr, CAD, Hypertension, PostopVasopressor, Age, CHF, Steroid, BMI, PostopRR, Stroke, CCB, Cirrhosis, Crystalloid, Cancer, BloodLoss, Anticoagulant, OSA |
| 31 | CharlsonIndex, Insulin, Sodium, ObesityGrade, Diabetes, IntraopMinHR, BetaBlocker, HbA1c, Arrhythmia, COPD, AntiPlatelet, CKD, ACEI_ARB, Diuretic, Cr, CAD, Hypertension, PostopVasopressor, Age, CHF, Steroid, BMI, PostopRR, Stroke, CCB, Cirrhosis, Crystalloid, Cancer, BloodLoss, Anticoagulant, OSA |
| 30 | Insulin, Sodium, ObesityGrade, Diabetes, IntraopMinHR, BetaBlocker, HbA1c, Arrhythmia, COPD, AntiPlatelet, CKD, ACEI_ARB, Diuretic, Cr, CAD, Hypertension, PostopVasopressor, Age, CHF, Steroid, BMI, PostopRR, Stroke, CCB, Cirrhosis, Crystalloid, Cancer, BloodLoss, Anticoagulant, OSA |
| 29 | Sodium, ObesityGrade, Diabetes, IntraopMinHR, BetaBlocker, HbA1c, Arrhythmia, COPD, AntiPlatelet, CKD, ACEI_ARB, Diuretic, Cr, CAD, Hypertension, PostopVasopressor, Age, CHF, Steroid, BMI, PostopRR, Stroke, CCB, Cirrhosis, Crystalloid, Cancer, BloodLoss, Anticoagulant, OSA |
| 28 | ObesityGrade, Diabetes, IntraopMinHR, BetaBlocker, HbA1c, Arrhythmia, COPD, AntiPlatelet, CKD, ACEI_ARB, Diuretic, Cr, CAD, Hypertension, PostopVasopressor, Age, CHF, Steroid, BMI, PostopRR, Stroke, CCB, Cirrhosis, Crystalloid, Cancer, BloodLoss, Anticoagulant, OSA |
| 27 | Diabetes, IntraopMinHR, BetaBlocker, HbA1c, Arrhythmia, COPD, AntiPlatelet, CKD, ACEI_ARB, Diuretic, Cr, CAD, Hypertension, PostopVasopressor, Age, CHF, Steroid, BMI, PostopRR, Stroke, CCB, Cirrhosis, Crystalloid, Cancer, BloodLoss, Anticoagulant, OSA |
| 26 | IntraopMinHR, BetaBlocker, HbA1c, Arrhythmia, COPD, AntiPlatelet, CKD, ACEI_ARB, Diuretic, Cr, CAD, Hypertension, PostopVasopressor, Age, CHF, Steroid, BMI, PostopRR, Stroke, CCB, Cirrhosis, Crystalloid, Cancer, BloodLoss, Anticoagulant, OSA |
| 25 | BetaBlocker, HbA1c, Arrhythmia, COPD, AntiPlatelet, CKD, ACEI_ARB, Diuretic, Cr, CAD, Hypertension, PostopVasopressor, Age, CHF, Steroid, BMI, PostopRR, Stroke, CCB, Cirrhosis, Crystalloid, Cancer, BloodLoss, Anticoagulant, OSA |
| 24 | HbA1c, Arrhythmia, COPD, AntiPlatelet, CKD, ACEI_ARB, Diuretic, Cr, CAD, Hypertension, PostopVasopressor, Age, CHF, Steroid, BMI, PostopRR, Stroke, CCB, Cirrhosis, Crystalloid, Cancer, BloodLoss, Anticoagulant, OSA |
| 23 | Arrhythmia, COPD, AntiPlatelet, CKD, ACEI_ARB, Diuretic, Cr, CAD, Hypertension, PostopVasopressor, Age, CHF, Steroid, BMI, PostopRR, Stroke, CCB, Cirrhosis, Crystalloid, Cancer, BloodLoss, Anticoagulant, OSA |
| 22 | COPD, AntiPlatelet, CKD, ACEI_ARB, Diuretic, Cr, CAD, Hypertension, PostopVasopressor, Age, CHF, Steroid, BMI, PostopRR, Stroke, CCB, Cirrhosis, Crystalloid, Cancer, BloodLoss, Anticoagulant, OSA |
| 21 | AntiPlatelet, CKD, ACEI_ARB, Diuretic, Cr, CAD, Hypertension, PostopVasopressor, Age, CHF, Steroid, BMI, PostopRR, Stroke, CCB, Cirrhosis, Crystalloid, Cancer, BloodLoss, Anticoagulant, OSA |
| 20 | CKD, ACEI_ARB, Diuretic, Cr, CAD, Hypertension, PostopVasopressor, Age, CHF, Steroid, BMI, PostopRR, Stroke, CCB, Cirrhosis, Crystalloid, Cancer, BloodLoss, Anticoagulant, OSA |
| 19 | ACEI_ARB, Diuretic, Cr, CAD, Hypertension, PostopVasopressor, Age, CHF, Steroid, BMI, PostopRR, Stroke, CCB, Cirrhosis, Crystalloid, Cancer, BloodLoss, Anticoagulant, OSA |
| 18 | Diuretic, Cr, CAD, Hypertension, PostopVasopressor, Age, CHF, Steroid, BMI, PostopRR, Stroke, CCB, Cirrhosis, Crystalloid, Cancer, BloodLoss, Anticoagulant, OSA |
| 17 | Cr, CAD, Hypertension, PostopVasopressor, Age, CHF, Steroid, BMI, PostopRR, Stroke, CCB, Cirrhosis, Crystalloid, Cancer, BloodLoss, Anticoagulant, OSA |
| 16 | CAD, Hypertension, PostopVasopressor, Age, CHF, Steroid, BMI, PostopRR, Stroke, CCB, Cirrhosis, Crystalloid, Cancer, BloodLoss, Anticoagulant, OSA |
| 15 | Hypertension, PostopVasopressor, Age, CHF, Steroid, BMI, PostopRR, Stroke, CCB, Cirrhosis, Crystalloid, Cancer, BloodLoss, Anticoagulant, OSA |
| 14 | PostopVasopressor, Age, CHF, Steroid, BMI, PostopRR, Stroke, CCB, Cirrhosis, Crystalloid, Cancer, BloodLoss, Anticoagulant, OSA |
| 13 | Age, CHF, Steroid, BMI, PostopRR, Stroke, CCB, Cirrhosis, Crystalloid, Cancer, BloodLoss, Anticoagulant, OSA |
| 12 | CHF, Steroid, BMI, PostopRR, Stroke, CCB, Cirrhosis, Crystalloid, Cancer, BloodLoss, Anticoagulant, OSA |
| 11 | Steroid, BMI, PostopRR, Stroke, CCB, Cirrhosis, Crystalloid, Cancer, BloodLoss, Anticoagulant, OSA |
| 10 | BMI, PostopRR, Stroke, CCB, Cirrhosis, Crystalloid, Cancer, BloodLoss, Anticoagulant, OSA |
| 9 | PostopRR, Stroke, CCB, Cirrhosis, Crystalloid, Cancer, BloodLoss, Anticoagulant, OSA |
| 8 | Stroke, CCB, Cirrhosis, Crystalloid, Cancer, BloodLoss, Anticoagulant, OSA |
| 7 | CCB, Cirrhosis, Crystalloid, Cancer, BloodLoss, Anticoagulant, OSA |
| 6 | Cirrhosis, Crystalloid, Cancer, BloodLoss, Anticoagulant, OSA |
| 5 | Crystalloid, Cancer, BloodLoss, Anticoagulant, OSA |
| 4 | Cancer, BloodLoss, Anticoagulant, OSA |
| 3 | BloodLoss, Anticoagulant, OSA |
| 2 | Anticoagulant, OSA |
| 1 | OSA |
