# Supplementary material for: An interpretable machine learning model for predicting postoperative hypotension in type 2 diabetes mellitus undergoing non‑cardiac surgery
Source: Cardiovasc Diabetol. 2026 Jun 22;25:265. doi: 10.1186/s12933-026-03256-3 (PMC13536729; doi:10.1186/s12933-026-03256-3)
Supplement: Supplementary file 16 [file 12933_2026_3256_MOESM6_ESM.docx]

Supplementary Table S5. Comprehensive performance of machine learning models on the training set with 95% confidence intervals for AUC.

| Model | AUC | 95% CI for AUC | Sensitivity | Specificity | Accuracy | Precision | F1‑Score | Brier Score |
| --- | --- | --- | --- | --- | --- | --- | --- | --- |
| Random Forest | 0.8430 | 0.837–0.849 | 0.9318 | 0.4932 | 0.7546 | 0.8305 | 0.6189 | 0.1699 |
| KNN | 0.8083 | 0.801–0.816 | 0.8287 | 0.6054 | 0.7385 | 0.7055 | 0.6516 | 0.1717 |
| XGBoost | 0.7544 | 0.746–0.763 | 0.8435 | 0.4908 | 0.7011 | 0.6801 | 0.5702 | 0.1961 |
| Neural Network | 0.7464 | 0.738–0.755 | 0.8117 | 0.5230 | 0.6951 | 0.6530 | 0.5808 | 0.1974 |
| Naïve Bayes | 0.7461 | 0.738–0.754 | 0.9999 | 0.0005 | 0.5962 | 0.8750 | 0.0011 | 0.3497 |
| Logistic Regression | 0.7455 | 0.737–0.754 | 0.8166 | 0.5129 | 0.6939 | 0.6547 | 0.5752 | 0.1977 |
| GLM | 0.7455 | 0.737–0.754 | 0.8166 | 0.5129 | 0.6939 | 0.6547 | 0.5752 | 0.1977 |
| LDA | 0.7455 | 0.737–0.754 | 0.8195 | 0.5096 | 0.6943 | 0.6567 | 0.5739 | 0.1978 |
| Elastic Net | 0.7455 | 0.737–0.754 | 0.8250 | 0.5019 | 0.6945 | 0.6602 | 0.5703 | 0.1979 |
| LASSO | 0.7454 | 0.737–0.754 | 0.8184 | 0.5107 | 0.6941 | 0.6559 | 0.5743 | 0.1978 |
| AdaBoost | 0.7441 | 0.736–0.752 | 0.8140 | 0.5156 | 0.6934 | 0.6526 | 0.5761 | 0.2009 |
| GBDT | 0.7438 | 0.736–0.752 | 0.8454 | 0.4678 | 0.6929 | 0.6723 | 0.5517 | 0.2010 |
| GBM | 0.7395 | 0.731–0.748 | 0.8501 | 0.4556 | 0.6907 | 0.6731 | 0.5434 | 0.2030 |
| Decision Tree | 0.6332 | 0.624–0.642 | 0.8079 | 0.4126 | 0.6483 | 0.5928 | 0.4866 | 0.2251 |
| Footnote: 95% confidence intervals for AUC were calculated using bootstrap resampling with 1,000 replicates. For each replicate, the training set was sampled with replacement, the model was retrained, and AUC was computed on the original training set (or via cross‑validation). The 2.5th and 97.5th percentiles of the bootstrap distribution are reported. | | | | | | | | |
