# Supplementary material for: An interpretable machine learning model for predicting postoperative hypotension in type 2 diabetes mellitus undergoing non‑cardiac surgery
Source: Cardiovasc Diabetol. 2026 Jun 22;25:265. doi: 10.1186/s12933-026-03256-3 (PMC13536729; doi:10.1186/s12933-026-03256-3)
Supplement: Supplementary file 17 [file 12933_2026_3256_MOESM7_ESM.docx]

Supplementary Table S6. Detailed performance metrics of different stacking ensemble model combinations on the training set with 95% confidence intervals for AUC.

| Model | AUC | 95% CI for AUC | Sensitivity | Specificity | Accuracy | Precision | F1‑Score | Brier Score | Combination Size | Combination |
| --- | --- | --- | --- | --- | --- | --- | --- | --- | --- | --- |
| Stacking_Ensemble | 0.7188 | 0.712–0.726 | 0.8136 | 0.4735 | 0.6762 | 0.6326 | 0.5416 | 0.2067 | 2 | RandomForest, KNN |
| Stacking_Ensemble | 0.7188 | 0.712–0.726 | 0.8122 | 0.4760 | 0.6764 | 0.6321 | 0.5431 | 0.2070 | 2 | RandomForest, XGBoost |
| Stacking_Ensemble | 0.7212 | 0.714–0.728 | 0.8081 | 0.4875 | 0.6786 | 0.6326 | 0.5506 | 0.2064 | 2 | RandomForest, NeuralNetwork |
| Stacking_Ensemble | 0.7234 | 0.716–0.731 | 0.8316 | 0.4557 | 0.6797 | 0.6471 | 0.5348 | 0.2056 | 2 | RandomForest, NaiveBayes |
| Stacking_Ensemble | 0.7152 | 0.708–0.722 | 0.8136 | 0.4659 | 0.6732 | 0.6288 | 0.5353 | 0.2081 | 2 | KNN, XGBoost |
| Stacking_Ensemble | 0.7055 | 0.698–0.713 | 0.8061 | 0.4637 | 0.6678 | 0.6184 | 0.5300 | 0.2112 | 2 | KNN, NeuralNetwork |
| Stacking_Ensemble | 0.7168 | 0.709–0.724 | 0.8608 | 0.4008 | 0.6750 | 0.6612 | 0.4991 | 0.2081 | 2 | KNN, NaiveBayes |
| Stacking_Ensemble | 0.7185 | 0.711–0.726 | 0.8120 | 0.4742 | 0.6755 | 0.6309 | 0.5414 | 0.2072 | 2 | XGBoost, NeuralNetwork |
| Stacking_Ensemble | 0.7223 | 0.715–0.730 | 0.8443 | 0.4299 | 0.6769 | 0.6517 | 0.5180 | 0.2064 | 2 | XGBoost, NaiveBayes |
| Stacking_Ensemble | 0.7255 | 0.718–0.733 | 0.8518 | 0.4256 | 0.6796 | 0.6606 | 0.5177 | 0.2058 | 2 | NeuralNetwork, NaiveBayes |
| Stacking_Ensemble | 0.7238 | 0.716–0.731 | 0.8169 | 0.4814 | 0.6814 | 0.6406 | 0.5497 | 0.2053 | 3 | RandomForest, KNN, XGBoost |
| Stacking_Ensemble | 0.7237 | 0.716–0.731 | 0.8081 | 0.4909 | 0.6800 | 0.6342 | 0.5534 | 0.2055 | 3 | RandomForest, KNN, NeuralNetwork |
| Stacking_Ensemble | 0.7238 | 0.716–0.731 | 0.8297 | 0.4526 | 0.6774 | 0.6430 | 0.5313 | 0.2059 | 3 | RandomForest, KNN, NaiveBayes |
| Stacking_Ensemble | 0.7233 | 0.716–0.731 | 0.8085 | 0.4905 | 0.6801 | 0.6345 | 0.5533 | 0.2055 | 3 | RandomForest, XGBoost, NeuralNetwork |
| Stacking_Ensemble | 0.7300 | 0.723–0.737 | 0.8358 | 0.4620 | 0.6848 | 0.6560 | 0.5421 | 0.2035 | 3 | RandomForest, XGBoost, NaiveBayes |
| Stacking_Ensemble | 0.7279 | 0.720–0.735 | 0.8304 | 0.4652 | 0.6829 | 0.6503 | 0.5424 | 0.2041 | 3 | RandomForest, NeuralNetwork, NaiveBayes |
| Stacking_Ensemble | 0.7193 | 0.712–0.727 | 0.8074 | 0.4805 | 0.6753 | 0.6283 | 0.5446 | 0.2070 | 3 | KNN, XGBoost, NeuralNetwork |
| Stacking_Ensemble | 0.7211 | 0.714–0.728 | 0.8378 | 0.4385 | 0.6765 | 0.6469 | 0.5227 | 0.2063 | 3 | KNN, XGBoost, NaiveBayes |
| Stacking_Ensemble | 0.7183 | 0.711–0.726 | 0.8541 | 0.4206 | 0.6790 | 0.6615 | 0.5142 | 0.2073 | 3 | KNN, NeuralNetwork, NaiveBayes |
| Stacking_Ensemble | 0.7239 | 0.716–0.732 | 0.8280 | 0.4607 | 0.6796 | 0.6448 | 0.5374 | 0.2058 | 3 | XGBoost, NeuralNetwork, NaiveBayes |
| Stacking_Ensemble | 0.7223 | 0.715–0.730 | 0.8115 | 0.4828 | 0.6787 | 0.6345 | 0.5483 | 0.2060 | 4 | RandomForest, KNN, XGBoost, NeuralNetwork |
| Stacking_Ensemble | 0.7275 | 0.720–0.735 | 0.8353 | 0.4595 | 0.6835 | 0.6540 | 0.5398 | 0.2043 | 4 | RandomForest, KNN, XGBoost, NaiveBayes |
| Stacking_Ensemble | 0.7233 | 0.716–0.731 | 0.8263 | 0.4668 | 0.6811 | 0.6456 | 0.5418 | 0.2056 | 4 | RandomForest, KNN, NeuralNetwork, NaiveBayes |
| Stacking_Ensemble | 0.7221 | 0.715–0.729 | 0.8224 | 0.4641 | 0.6777 | 0.6391 | 0.5378 | 0.2059 | 4 | RandomForest, XGBoost, NeuralNetwork, NaiveBayes |
| Stacking_Ensemble | 0.7313 | 0.724–0.739 | 0.8366 | 0.4595 | 0.6843 | 0.6559 | 0.5404 | 0.2030 | 4 | KNN, XGBoost, NeuralNetwork, NaiveBayes |
| Footnote: 95% confidence intervals for AUC were calculated using bootstrap resampling with 1,000 replicates. For each replicate, the training set was sampled with replacement, the stacking ensemble model was retrained, and AUC was computed on the original training set (or via cross‑validation). The 2.5th and 97.5th percentiles of the bootstrap distribution are reported. The narrow CI widths (approximately ±0.003 to ±0.005) reflect the large training sample size (n=23,843) and stable model performance. This method was consistently applied to all stacking combinations. | | | | | | | | | | |
